# TBX3 regulates the transcription of VEGFA to promote osteoblasts proliferation and microvascular regeneration

**DOI:** 10.7717/peerj.13722

**Published:** 2022-07-11

**Authors:** Lichuang Wu, Chenxian Su, Chuanhua Yang, Jinxing Liu, Yiheng Ye

**Affiliations:** 1 Department of Trauma/Joint Surgery, The First Affiliated Hospital of Wenzhou Medical University, Wenzhou, China; 2 The Affiliated Hospital of Shandong University of Traditional Chinese Medicine, Shandong, China; 3 Shanghai ninth people’s Hospital, Shanghai Jiao Tong University School of Medicine, Shanghai, China

**Keywords:** Osteogenesis, VEGF, Regulon, TBX3, Vascularization, Osteoblasts proliferation

## Abstract

**Objective:**

Osteochondral decellularization can promote local vascular regeneration, but the exact mechanism is unknown. The aim of this study is to study osteogenic microvascular regeneration in single cells.

**Methods:**

The scRNA-seq dataset of human periosteal-derived cells (hPDCs) were analyzed by pySCENIC. To examine the role of TBX3 in osteogenesis and vascularization, cell transfection, qRT-PCR, western blot, and CCK-8 cell proliferation assays were performed.

**Results:**

TCF7L2, TBX3, FLI1, NFKB2, and EZH2 were found to be transcription factors (TFs) most closely associated with corresponding cells. The regulatory network of these TFs was then visualized. Our study knocked down the expression of TBX3 in human osteoblast cell lines. In the TBX3 knockdown group, we observed decreased expression of VEGFA, VEGFB, and VEGFC. Moreover, Western blot analysis showed that downregulating TBX3 resulted in a reduction of VEGFA expression. And TBX3 stimulated osteoblast proliferation in CCK-8 assays.

**Conclusion:**

TBX3 regulates VEGFA expression and promotes osteoblast proliferation in skeletal microvasculature formation. The findings provide a theoretical basis for investigating the role of TBX3 in promoting local vascular regeneration.

## Introduction

The human body consists of several systems, and the skeletal-muscular system provides its physical framework, which provides movement, support, and stability. As a result of pathological conditions, such as osteoporosis, bone fractures, joint deformities, bone softening, or bone tumors, the structure of bones may change, causing severe consequences. Increasing age decreases the repair capacity of bones, resulting in an increased risk of developing pathological bone conditions (Reid & Billington, 2022; Soós et al., 2022; Cui et al., 2022).

A recent study has shown that both osteoblasts and osteoclasts mediate bone repair (Saul & Khosla, 2022). A dynamic balance between the two is key to the repair capacity, with osteoblasts playing an essential function during bone formation (Saul & Khosla, 2022). Osteoblasts secrete type I collagen, bone bridging protein, alkaline phosphatase, and other proteins (Cui et al., 2022). Osteoblasts undergo three stages of differentiation, including osteogenic progenitor cells, preosteoblasts, and osteoblasts. The Wnt/*β*-catenin signalling pathway plays an essential role in transforming preosteoblasts into osteoblasts inducing the expression of the osteoblast marker osterix (Mora-Raimundo et al., 2019; Zhou et al., 2021). Under pathological conditions, osteoblasts undergo mechanical stress, and the expression of Dickkopf-related protein 1 (DKK1) and sclerostin are reduced, resulting in increased wnt/*β*-catenin signaling and osteoblast activation, thereby promoting bone repair (Li et al., 2022b; Chico et al., 2022). IL-1R1/MyD88 signalling pathway is involved in the negative regulation of bone repair (Martino et al., 2016).

During bone repair, the microenvironment of bone plays an important role, where the microvasculature formation can accelerate bone repair and healing. The formation of a microvascular network is necessary for bone regeneration, and insufficient angiogenesis may delay it (Colnot & Helms, 2001). Vascular endothelial growth factor (VEGF) is a highly specific pro-vascular endothelial growth factor essential for angiogenesis (Zeng et al., 2019) and is regulated by a variety of transcription factors (TFs), including SP1 (Liu et al., 2016), HIF-1 (Li et al., 2021; Campochiaro, 2015), and STAT3 (Jiang et al., 2011, p. 3). These TFs not only bind directly to the VEGF promoter but also act synergistically with other factors, including growth factors, to regulate gene transcription (Arcondeguy et al., 2013). Therefore, we hypothesize that TFs influence microangiogenesis during bone repair by regulating VEGF expression.

In single gene sequencing, transcriptome data can be analyzed at the cellular level. We screened TFs that might be involved in regulating VEGF+ cell populations by using single-cell sequencing data. A molecular regulatory mechanism is implicated in skeletal microvascular regeneration in our study.

## Methods

### Data quality control and pre-processing

Single-cell RNA sequencing data were downloaded from sample GSM4119369 (from the GSE138791 dataset) of human periosteal-derived cells (hPDCs) and treated in a serum-free growth medium for six days (Bolander et al., 2020). Low-quality cells were defined as follows: (1) cells with less than 200 genes detected; (2) cells with >4,000 genes detected; and (3) cells with more than 5% mitochondrial genes. Low-quality cells are usually considered non-viable cells and therefore removed from the dataset. Finally, we screened 11,119 cells and 16,884 genes.

Gene expression data were normalized and scaled using the Seurat (Version 4.1.0) package (Hao et al., 2021). The data were normalized using the “NormalizeData” function, the normalization method was set to “LogNormalize”, and the scaling factor was 100000. Then, the “FindVariableFeatures” function was used to filter the first 2000 high-variable genes, and the subsequent downscaling to these genes was restricted. Further, the “RunPCA” function was used to perform principal component analysis (PCA) on the single-cell expression matrix restricted to the highly variable genes. The signal-to-noise ratio was improved by selecting partially significant principal components. The significance of each principal component was calculated using the “JackStraw” method provided in the Seurat package, and the top 40 principal components were selected for subsequent analysis.

Cell clustering analyses were done using the “FindClusters” function, with the resolution set to 1.5, and by embedding cells into the graph structure in PCA space. Subsequently, RunUMAP and RunTSNE functions were used to perform UMAP and tSNE dimensionality reduction analysis.

### Cell type annotation

Cell subgroups were annotated according to the expression levels and ratios of marker genes in the clustered subgroups. The marker genes corresponding to the cell types were: mitotic cell - MKI67, AURKA, CDC20, CDK1; pro-hematopoietic osteochondral progenitor- ANGPTL7; BMPR2+ osteochondral progenitor—POSTN, BMPR2; CD34+ osteochondral progenitor—POSTN, CD34; Osteochondral progenitor—POSTN, PDGFRB, THY1.

### Gene pooling enrichment analysis

Gene Ontology (GO) provides a framework to describe the function of gene products in all organisms. GO annotations include three major categories: Biological Process (BP), Cellular Component (CC), and Molecular Function (MF). The KEGG knowledge base collects a large amount of manually proofread information on biological pathway networks. To enrich the set of the gene of interest into GO terms and KEGG terms, the “enrichGO” and “enrichKEGG” functions in the clusterProfiler package were used, respectively (Version 4.2.2; Yu et al., 2012; Wu et al., 2021). In addition, the “GSEA” function was used to determine whether the pathway gene sets were significantly different between the two biological states, and an FDR < 0.05 was set as significant enrichment.

### Transcriptional regulation analysis

To infer the gene regulatory networks of VEGF+ cell subpopulations, the SCENIC (Version 1.2.4, Aibar et al., 2017) toolkit was used. SCENIC consisted of three steps: (1) Identification of co-expression modules between TFs and potential target genes; (2) for each co-expression module, identification of the direct target of the TF by TF-motif enrichment analysis genes, and grouping of each TF and its corresponding direct target gene into a regulon; (3) scoring each regulon activity for every cell using the AUCell algorithm (AUC).

To quantify the cell type specificity of regulon, a pre-described algorithm was used (Suo et al., 2018). A vector to represent the distribution of the activity fraction of a given regulon in the cell population was used. Then its activity was normalized to a sum of 1. Moreover, to indicate whether each cell belongs to a particular type, a vector was set with its elements taking values of 0 or 1, with 1 indicating that the cell belongs to a specific cell type and 0 suggesting that it did not. We also normalized this vector to a sum of 1. Next, we calculated the Jensen–Shannon scatter (JSD) between these two vectors, often used to quantify the difference between two probability distributions. The JSD values range from 0 to 1, 0 indicating the same distribution and 1 indicating a significant difference. Finally, the modulator specificity score (RSS) was defined by converting the JSD to a similarity score.

The smaller the JSD, the larger the RSS. Significant regulators were defined as those with the highest cell type specificity score for each cell type. Cytoscape (Version 3.8.0) was to visualize the regulatory-related networks of target cells.

### Regulation module analysis

Transcriptional regulatory modules were identified using the Conjugation Specificity Index (CSI). The calculation of CSI consisted of two steps. First, the Pearson correlation coefficient (PCC) between the activity scores of each pair of regulators was calculated. Next, the Euclidean distance hierarchical clustering method based on the CSI matrix was used to identify different regulatory modules. For each regulatory module, the activity score for a cell type was defined as the average of the activity scores of its regulatory members across all cells of that type. The top-ranked cell types were then identified for each module.

### Cell culture and transfection

The human osteoblast cell line was purchased from the Institute of Cell Research, Chinese Academy of Sciences, Shanghai, China, and cultured in DMEM high sugar medium, 10% FBS, and 1% double-antibody in a 5% CO_2_ constant temperature and humidity incubator. After the cells were treated with TBX3 expression intervention, the cells were transfected with 100 pmol TBX3 siRNA, and total cellular RNA was extracted after 48 h of treatment.

### Total cellular RNA extraction and qRT-PCR analysis

Total cellular RNA was extracted with Trizol (Beyotime, Shanghai, China) reagent, and the experiments were performed according to the commercial instructions. The extracted RNA was quantified by NanoDrop and reverse transcribed into cDNA by GoScript™ Reverse Transcription Kit (Promega, Wisconsin, USA), followed by FastFire qPCR PreMix (SYBR Green, TIANGEN, China) to determine the expression level of mRNA. The mRNA expression levels were calculated as 2^−ΔΔCt,^ and GAPDH was used as an internal reference.

### Protein extraction and western blot

Total protein was extracted by RIPA according to the manufacturers’ protocol, containing protease inhibitors. Equal amounts of protein were separated by 10% SDS-PAGE gel and transferred to PVDF membranes under certain conditions. The primary antibody was then incubated overnight under 4 °C, the next day, the secondary antibody was incubated for 1–2 h at room temperature, followed by exposure to developer and storage of the images through Tanon 5200 (Tanon, China). The primary antibodies used above include anti-VEGFA (1:1000, abcam, USA) and anti-GAPDH (1:2000, Servicebio, Wuhan, China). Among them, GAPDH was used as an internal reference.

### CCK-8 cell proliferation assay

We used the CCK-8 assay to determine the proliferative capacity of the cells. We divided the cells of TBX3 expression intervention for 48 h into 96-well plates equally, with 1*10^3^ cells per well. And we applied CCK-8 for cell proliferation assay every other day for a total of 5 days. For CCK-8 assay, 10 µL of CCK-8 reagent was added to each well, and OD450 value was measured after 3 h.

### Statistical analysis

All experimental samples, from at least three independent experiments, were expressed as mean ± standard deviation. The Chi-square test was used for the statistical analysis of frequencies between two groups. Statistical differences between two independent samples were analyzed by *t*-test. *P* <0. 05 was considered statistically significant.

## Results

### Identification of VEGF+ cell subpopulations from human periosteal-derived cells (hPDCs)

Single-cell RNA sequencing data were acquired and processed (See ‘Methods’). After quality control and discarding low-quality cells, 11,119 hPDCs were used for further analysis (Figs. S1A–S1C). Based on the expression levels of marker genes in each clustered subgroup, the clustered subgroups were annotated as CD34+ osteochondral progenitor (*n* = 4563), osteochondral progenitor (*n* = 5316), pro −hematopoietic osteochondral progenitor (*n* = 600), BMPR2+ osteochondral progenitor (*n* = 534) and mitotic cells (*n* = 106, Fig. S1D–S1F).

**Figure 1:**
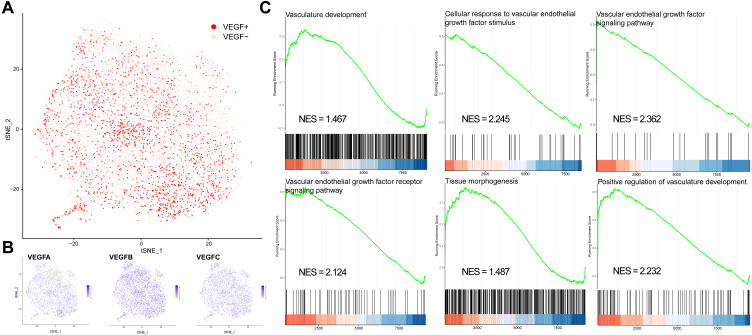
VEGF+ cell subpopulation localization. (A) Cells with positive expression of at least two of VEGFA, VEGFB, and VEGFC are classified as VEGF+ cells. (B) Scatter plot showing the distribution of VEGFA, VEGFB, and VEGFC expressions in all cells. (C) GSEA analysis showing the different pathways significantly upregulated in VEGF+ cells.

We classified all cells into VEGF+ cells (expressing at least two genes) and VEGF- cells (expressing at most one gene) based on the expression of VEGFA, VEGFB, and VEGFC (Figs. 1A–1B). VEGF+ cells were considered angiogenesis-related cells. We obtained 8697 VEGF- cells and 2422 VEGF+ cells. We next compared the significantly different GO and BP pathways between VEGF- and VEGF+ cells by gene pooling enrichment analysis. The results showed that pathways such as vascular vein development, VEGF-stimulated cellular responses, histogenesis, and VEGF pathways were significantly activated in VEGF+ cells (Fig. 1C), indicating that VEGF+ cells were indeed associated with an angiogenic activity.

### Reconstruction of transcriptional regulatory networks

To comprehensively reconstruct the gene regulatory network in a subpopulation of VEGF+ cells, we analyzed VEGF+ cell expression data using the SCENIC process. SCENIC linked cis-regulatory sequence information to single-cell RNA sequencing (scRNA-seq) data. SCENIC consists of three main steps: co-expression analysis, target gene motif enrichment analysis, and scoring of regulatory activity. The main results included a series of regulators (each representing a TF and a set of co-expressed and motif significantly enriched target genes) and a regulatory activity score (RAS) for each cell. The SCENIC analysis showed that out of the 274 regulators screened, 145 were activated in more than 1% of the cells (Table S1). We focused on those 145 transcriptional regulators. The number of target genes per regulator ranged from 10 to 918, involving 6,968 genes.

We calculated the specificity scores between the 145 regulons and different cell types separately to understand if regulons are specific to a particular cell type. We found that: TCF7L2 was most associated with CD34+ osteochondral progenitor; TBX3 was predominantly associated with osteochondral progenitor; FLI1 was most associated with pro-hematopoietic osteochondral progenitor; NFKB2 was most associated with BMPR2+ osteochondral progenitor and; EZH2 was most associated with Mitotic cells (Figs. S2A–S2E). We showed the distribution of different cell types with the corresponding most relevant regulon activated cells in TSNE space (Figs. S2A–S2E). TCF7L2 is mainly enriched in CD34+ osteochondral progenitor; TBX3 is mainly enriched in osteochondral progenitor; FLI1 is mainly enriched in pro-hematopoietic osteochondral progenitor; NFKB2 is mainly enriched in BMPR2+ osteochondral progenitor; and EZH2 is mainly enriched in mitotic cells.

**Figure 2:**
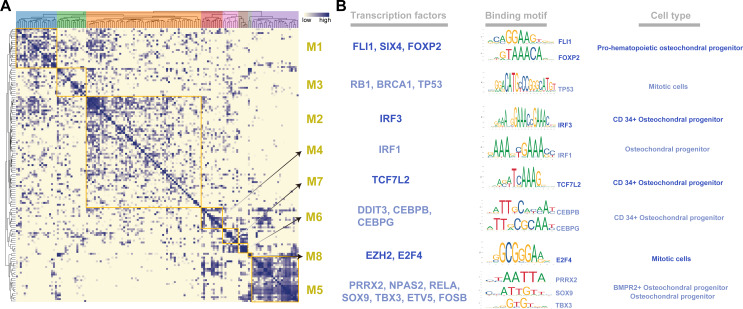
Identification of regulatory modules. (A) Heat map showing regulatory modules based on the Conjugation Specificity Indicator (CSI) matrix. (B) Single cell TF analysis showing motifs of different representative transcription factors and the cell types associated with the modules.

To systematically characterize the combination patterns among the 145 regulons, we compared the similarity between each regulon based on the CSI matrix and divided them into eight modules (M1- 21 regulons; M2- 59 regulons; M3- 15 regulons; M4- 11 regulons; M5- 24 regulons; M6- 5 regulons; M7- 8 regulons; M8- 2 regulons) (Fig. 2A). For each module, we identified several representative cell types by their average activity scores and the activity distribution of the modules in VEGF+ cells (Figs. S3A–S3B). These results demonstrate the presence of M1-M8 transcription factors in different cell types and individuals. In addition, we also list the TFs and their binding motifs that represented the cell type specificity within each module (Fig. 2B).

Finally, we used Cytoscape to visualize the regulatory network of important TFs (Fig. S4A). To explore which biological functions these TFs were mainly involved in, we performed GO and KEGG enrichment analysis on 145 TFs, showing the enrichment results of the top 10 KEGG pathways, BP, MF, and CC pathways that were significantly enriched (Figs. S4B– S4C). Pathway analysis showed that those TFs were involved mainly in transcriptional misregulation in cancer, hepatitis B, human T-cell leukemia virus 1 infection, viral carcinogenesis, kaposi sarcoma-associated herpesvirus infection, c-type lectin receptor signaling pathway, lipid and atherosclerosis, acute myeloid leukemia, hepatitis C, Th17 cell differentiation (*P* < 0.0001; Fig. S4B). Those TFs also enrichment in BPs (intracellular receptor signaling pathway, rhythmic process, pri-miRNA transcription by RNA polymerase II, mononuclear cell differentiation, anterior/posterior pattern specification, gland development, circadian rhythm, regionalization, and lymphocyte differentiation) MFs (DNA-binding transcription activator activity, DNA-binding transcription repressor activity, DNA-binding transcription factor binding, nuclear receptor activity, ligand-activated transcription factor activity, core promoter sequence-specific DNA binding, and chromatin DNA binding), and CCs (tracription regulator complex, RNA polymerase transcrintion regulator complex) (*P* < 0.0001; Fig. S4C). The results of these enrichment analyses clearly demonstrate the functions and pathways that are enriched by these TFs.

### TBX3 promoted the VEGF expression and cell proliferation

**Figure 3:**
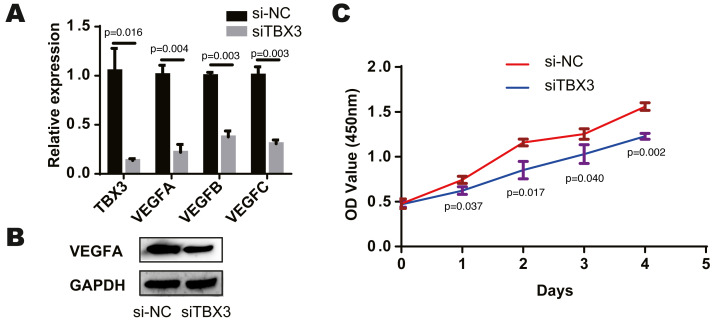
TBX3 promotes VEGF expression and cell proliferation. (A) qRT-PCR showing a decrease in VEGFA, VEGFB, and VEGFC after TBX3 knockdown. (B) Western blot images demonstrating a decrease in the expression of VEGFA after TBX3 knockdown. (C) CCK-8 assay showing TBX3 could reduce the proliferative ability of osteoblast cell lines. ^∗^*P* < 0.05, ^∗∗^*P* < 0.01, ^∗∗∗^*P* < 0.001.

We found that TCF7L2, TBX3, FLI1, NFKB2 and EZH2 were most closely related to the corresponding cells by bioinformatics prediction. According to the above study, TCF7L2, TBX3 and EZH2 are closely related to osteoblasts. And among the eight modules and cells, the osteochondral progenitor was closely associated with most of the modules. The TF most closely related to these cells was TBX3. We knocked down the expression of TBX3 in human osteoblast cell lines by treating them with a TBX3 downregulation. We then examined the expression of VEGFA, VEGFB, and VEGFC using qRT-PCR and found that their levels were decreased in the TBX3 knockdown group (Fig. 3A). Furthermore, the downregulation of TBX3 resulted in low expression of VEGFA by western blot (Fig. 3B). Subsequently, we examined the proliferation ability of the osteoblast cell line using CCK-8 and found that TBX3 could promote cell proliferation (Fig. 3C). Thus, we proposed that TBX3 is a transcription factor involved in skeletal microvasculature development by regulating VEGF expression and promoting osteoblast proliferation.

## Discussion

Many pathological conditions can lead to fractures, such as deformities, abnormal movements, and functional disorders, which negatively impact people’s quality of life (Beerman, Basisty & de Cabo, 2022). Osteoblasts and osteoclasts have the potential to repair fracture trauma on their own. However, the repair of fractures can be prolonged in various underlying diseases, such as diabetes (Chen et al., 2022b), vascular diseases (Liu et al., 2022), and autoimmune diseases (Tian et al., 2022). These diseases often affect bone repair by altering blood vessel formation, including microvascular regeneration (Zheng et al., 2022). In this study, TBX3 functions as a TF that regulates VEGF expression and has a positive effect on skeletal microvascular regeneration by regulating osteoblasts.

The VEGF family is an important class of molecules for blood vessel formation (Kumar et al., 2014), including VEGFA, VEGFB, VEGFC, and other proteins (Molema et al., 2022). It has been shown that VEGFA can maintain the normal functioning of the skeletal internal environment under physiological conditions. In pathological conditions, especially during bone repair, VEGFA promotes migration and proliferation of endothelial cells through BMP-2, thus accelerating bone repair (Bakshi et al., 2021; Chen et al., 2022a). Other studies showed that BMP-2 could act synergistically with VEGFA to promote bone repair through osteogenic and vascular pathways (Geng et al., 2021). VEGFB also has a similar enhancer region to VEGFA and can be regulated by homologous TFs (Silins et al., 1997). VEGFB can induce angiogenesis by activating Akt and eNOS-related pathways through receptor VEGFR-1 (Silvestre et al., 2003; Zhong et al., 2016) and also regulate microvascular regeneration (Ato et al., 2022). VEGFC, another form of the VEGF family protein, has a specific homologous region with VEGFA. It can be induced by TNF-α (Li et al., 2018) and improve diabetic foot by improving microvascular regeneration in diabetes (Zhou, Wei & He, 2021). These studies showed that VEGFA, VEGFB, and VEGFC could all be involved in vascular regeneration, including microvascular regeneration. This study screened the VEGF+ cell population using VEGFA, VEGFB, and VEGFC as markers. We used SCENIC to construct a transcriptional regulatory network and obtained the most closely related cytokines in the corresponding cells, including TCF7L2, TBX3, FLI1, NFKB2, and EZH2.

TCF7L2 is a key transcriptional effector molecule of the Wnt signalling pathway, which can affect diabetic nephropathy by regulating the expression of VEGFA (Luo et al., 2013). It can promote osteoblast differentiation through lipocalin 2 and also promote bone repair (Zhou et al., 2017; Yin et al., 2020, p. 2). It can be involved in osteoblast differentiation as a Wnt/ *β*-catenin signalling pathway. EZH2 (histone-lysine N-methyltransferase) is a widely studied TF that catalyzes lysine (H3K27) at position H327 of histone proteins. And it is a regulatory factor involved in regulating expression in the epigenome, and the transcriptom (Sun et al., 2022). Abnormal expression of EZH2 can cause neurodegenerative and neoplastic diseases (Li et al., 2013; Li et al., 2022a). Additionally, EZH2 affects osteoblast differentiation, proliferation, and epigenetic inheritance, which is associated with osteoblast differentiation (Galvan et al., 2021). Bone morphogenetic protein 2 (BMP2) and EZH2 can inhibit osteogenesis (Dudakovic et al., 2020, p. 2; Lui et al., 2021). On the other hand, EZH2 can also promote the osteogenesis of periodontal ligament stem cells through the wnt/ *β*-catenin pathway (Cheng & Zhou, 2020). Although different miRNAs and lncRNAs also promote osteogenesis by inhibiting EZH2 (Cai, Li & Wang, 2019, p. 2; Li et al., 2020, p. 2), their role in osteogenesis is currently unclear. Future studies should investigate the role of EZH2 TF during osteogenesis.

FLI1 encodes TFs containing the ETS region, primarily involved in diseases including Ewing sarcoma (Yoshimatsu et al., 2022, p. 1) and acute lymphoblastic leukaemia (Das et al., 2019). Similarly, NFKB2, a molecule of the classical NF-κB pathway, is closely associated with inflammation and immune regulation. It is involved in inflammatory diseases mainly by influencing the expression of inflammatory factor TBX3, containing an intrinsic protein block, T-BOX, which can regulate growth and development (Chawla et al., 2021). Mutations in the TBX3 gene can cause Ulnar-Mammary Syndrome and Holt-Oram Syndrome. On the one hand, TBX3 can promote the proliferation of adipose stromal stem cells and induce their differentiation into osteoblasts (Lee et al., 2007). On the other hand, it can negatively regulate osteoblast differentiation by inhibiting the expression of osterix and RUNX2, instead promoting osteoblast proliferation (Govoni et al., 2009, p. 2). Moreover, TBX3 can maintain the stability of umbilical vein endothelial cells by activating protein kinase B and VEGF (Yu et al., 2020). TBX3 is involved in limb and vascular development and can influence the proliferation and differentiation of osteoblasts. We divided the TFs into eight modules and found that the osteochondral progenitor was more closely related to each module. Upon further cellular studies on TBX3, we found that it promoted VEGF expression and osteoblast proliferation. In this study, TBX3 functions as a TF that regulates VEGF expression and has a positive effect on skeletal microvascular regeneration by regulating osteoblasts.

## Conclusion

In summary, this study obtained the molecular regulatory mechanism of the TBX3-VEGF regulatory axis. Our preliminary investigation indicates that TBX3 was a critical molecule regulating skeletal microvascular regeneration, providing a new molecular mechanism. Further *in vivo* and *ex vivo* experimental studies are needed to be performed in the future.

## Supplemental Information

10.7717/peerj.13722/supp-1 Supplemental Information 1 Quality control and cell-type annotation (A) Cell quality indicators. (B) Mean expression values and standard deviations of genes. (C) Principal component significance. (D) Cellular tSNE descending clustering. (E) Mean expression values and expression proportions of marker genes in clustered subgroups. (F) Distribution of different cell types includes: CD34+ osteochondral progenitor (*n* = 4563), osteochondral progenitor (*n* = 5316), pro-hematopoietic osteochondral progenitor (*n* = 600), BMPR2+ osteochondral progenitor (*n* = 534) and mitotic cells. (Related to Figs. 1A & 1B)

10.7717/peerj.13722/supp-2 Supplemental Information 2 Analysis of cell-type-specific regulator activity (A) The left panel indicates regulators activated in VEGF+ cells sorted according to specificity scoring with CD34+ osteochondral progenitor, with the top 5 regulators considered relevant. The middle panel highlights CD34+ osteochondral progenitor in all cells. The right panel is the fraction with the most specific regulator of CD34+ osteochondral progenitor, TCF7L2, activated in all VEGF+ cells. Like (B–E). (Related to Figs. 1A & 1B)

10.7717/peerj.13722/supp-3 Supplemental Information 3 Regulatory module activity analysis (A) Cell types were ranked according to their activity scoring with regulatory modules. (B) Activity scoring of regulatory modules in VEGF+ cells. (Related to Fig. 2)

10.7717/peerj.13722/supp-4 Supplemental Information 4 Transcriptional regulatory networks activation and enrichment analysis (A) Transcription factor regulatory networks activated in VEGF+ cells are shown. Deformed dots indicate regulatory factors, and pink dots indicate target genes. (B)Transcription factors activated in cells significantly enriched in the KEGG pathway of the top 10 pathways. (C) GSEA analysis showing the different pathways significantly upregulated in BP, MF, and CC. (Related to Fig. 2)

10.7717/peerj.13722/supp-5 Supplemental Information 5 Raw data for qRT-PCR & OD (Fig. 3)

10.7717/peerj.13722/supp-6 Supplemental Information 6 Full-length uncropped Western blots (Fig. 3)

10.7717/peerj.13722/supp-7 Supplemental Information 7 Transcriptional regulators

## Additional Information and Declarations

### Data Availability

The following information was supplied regarding data availability: The raw measurements are available in the Supplementary Files.
