# Supplementary figures and images for: TBX3 regulates the transcription of VEGFA to promote osteoblasts proliferation and microvascular regeneration
Source: PeerJ. 2022 Jul 11;10:e13722. doi: 10.7717/peerj.13722 (PMC9281600; doi:10.7717/peerj.13722)

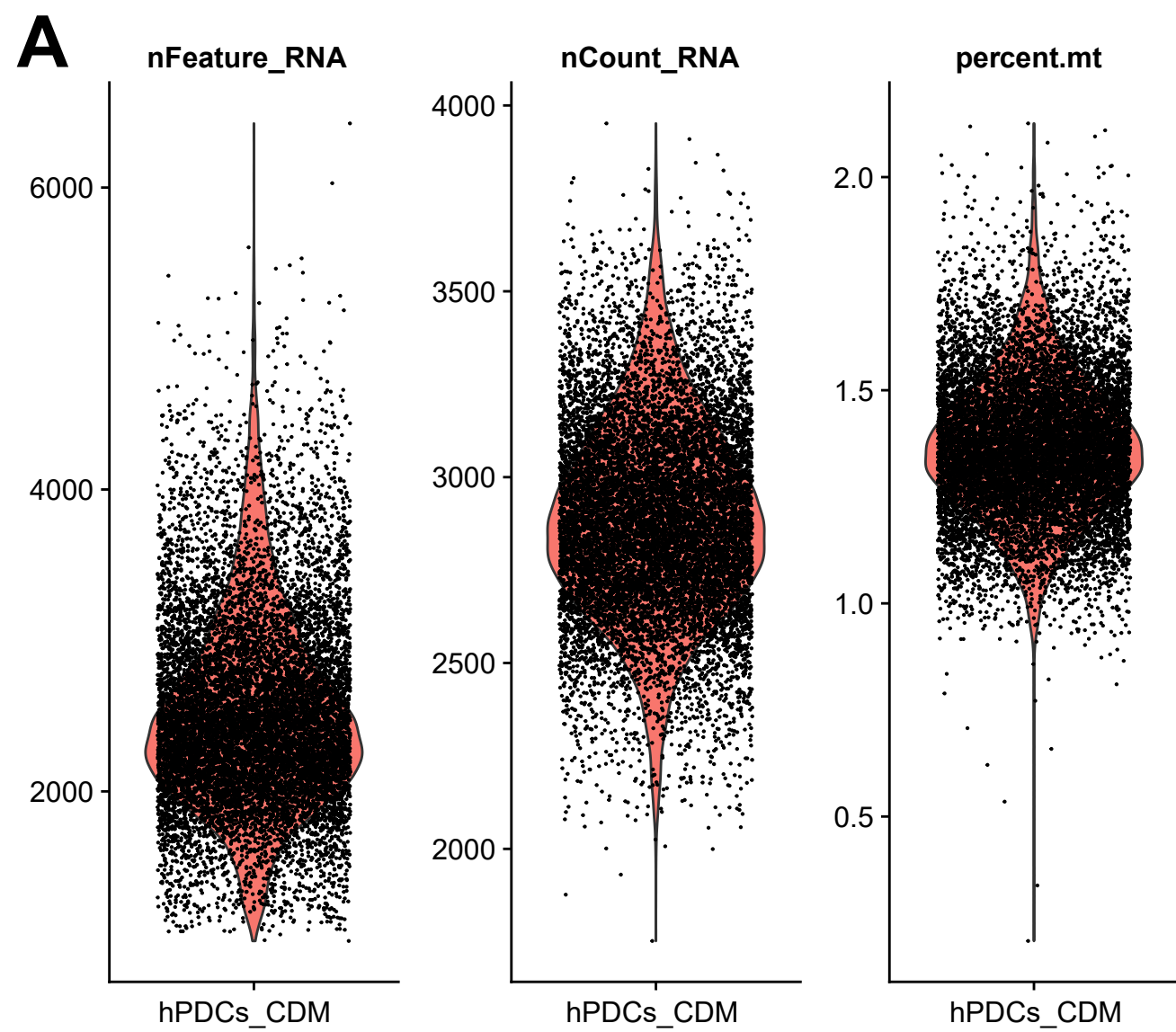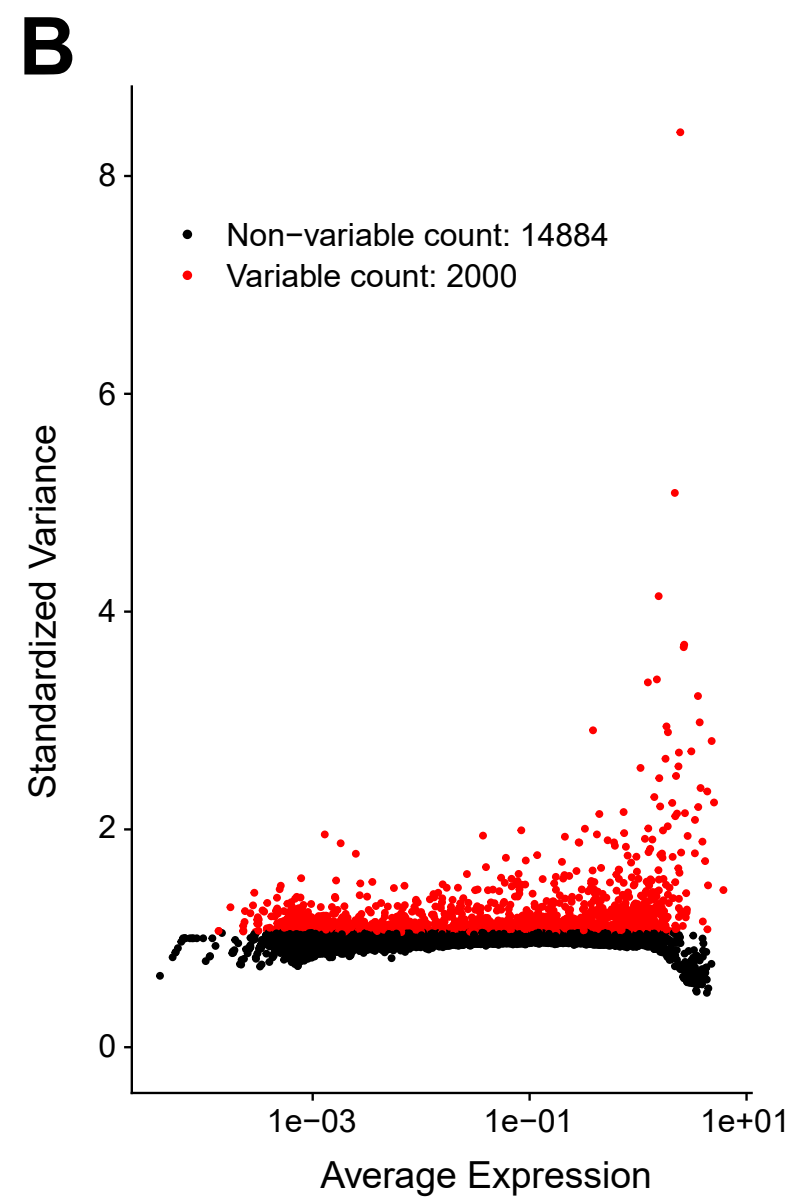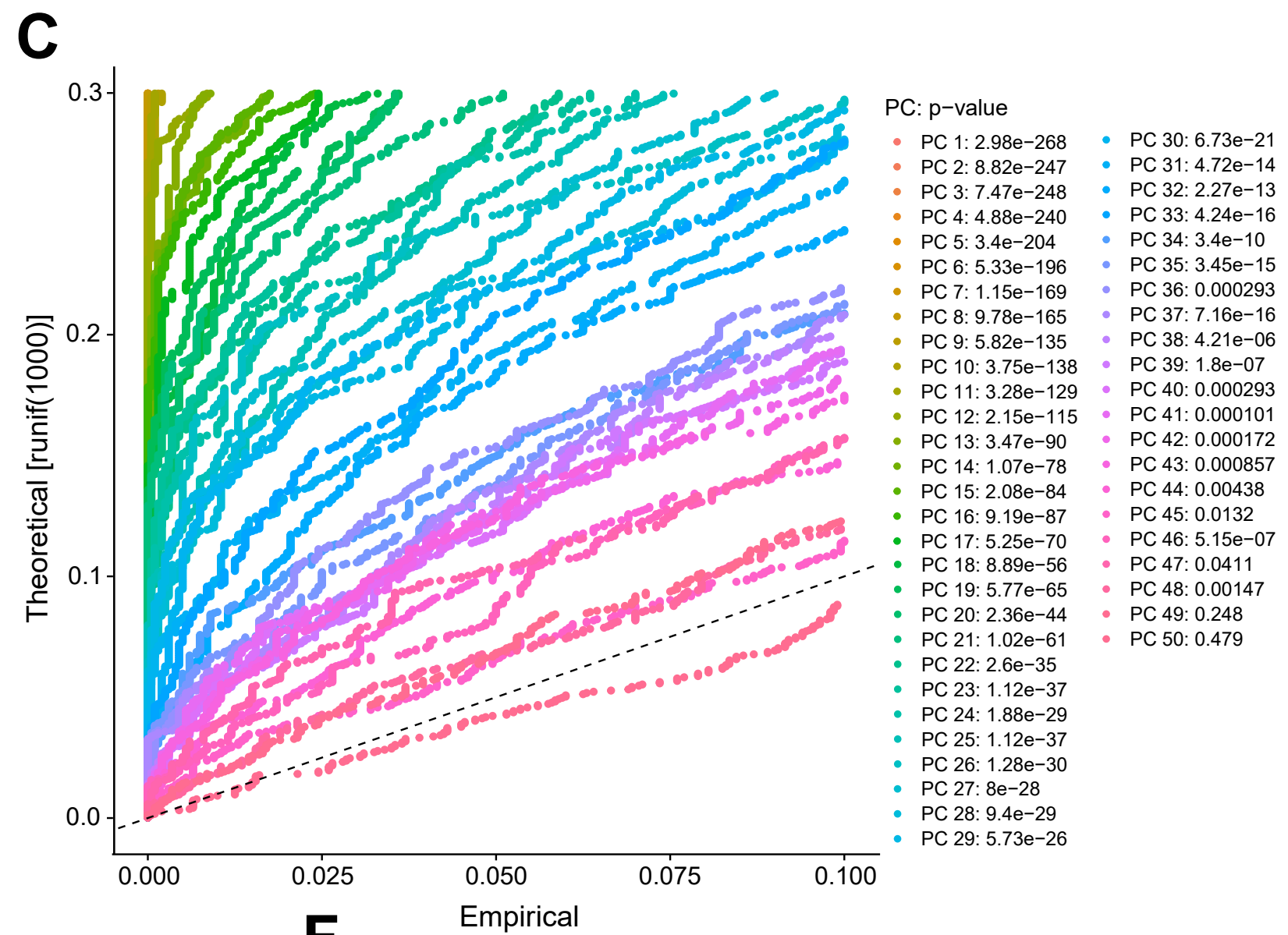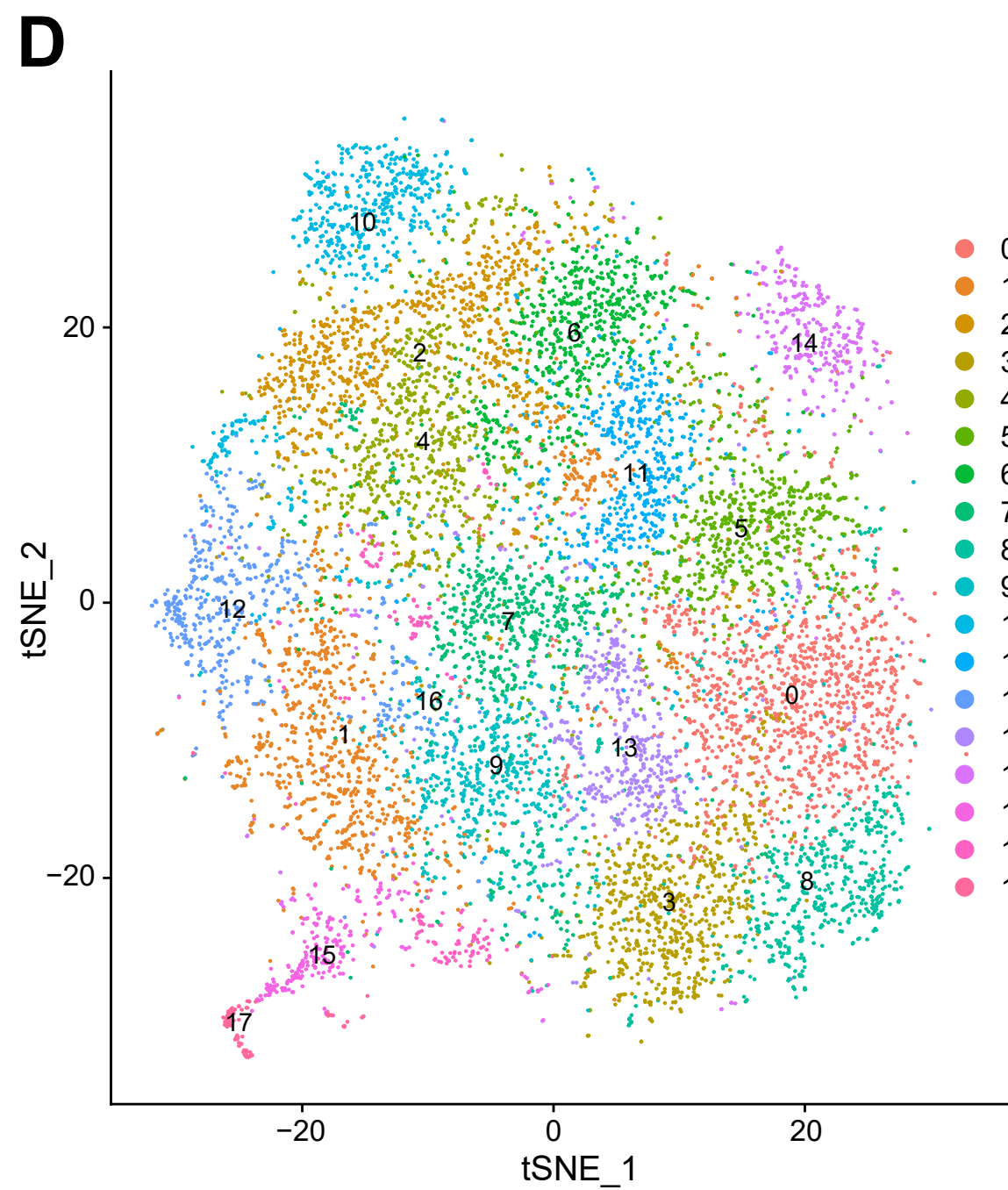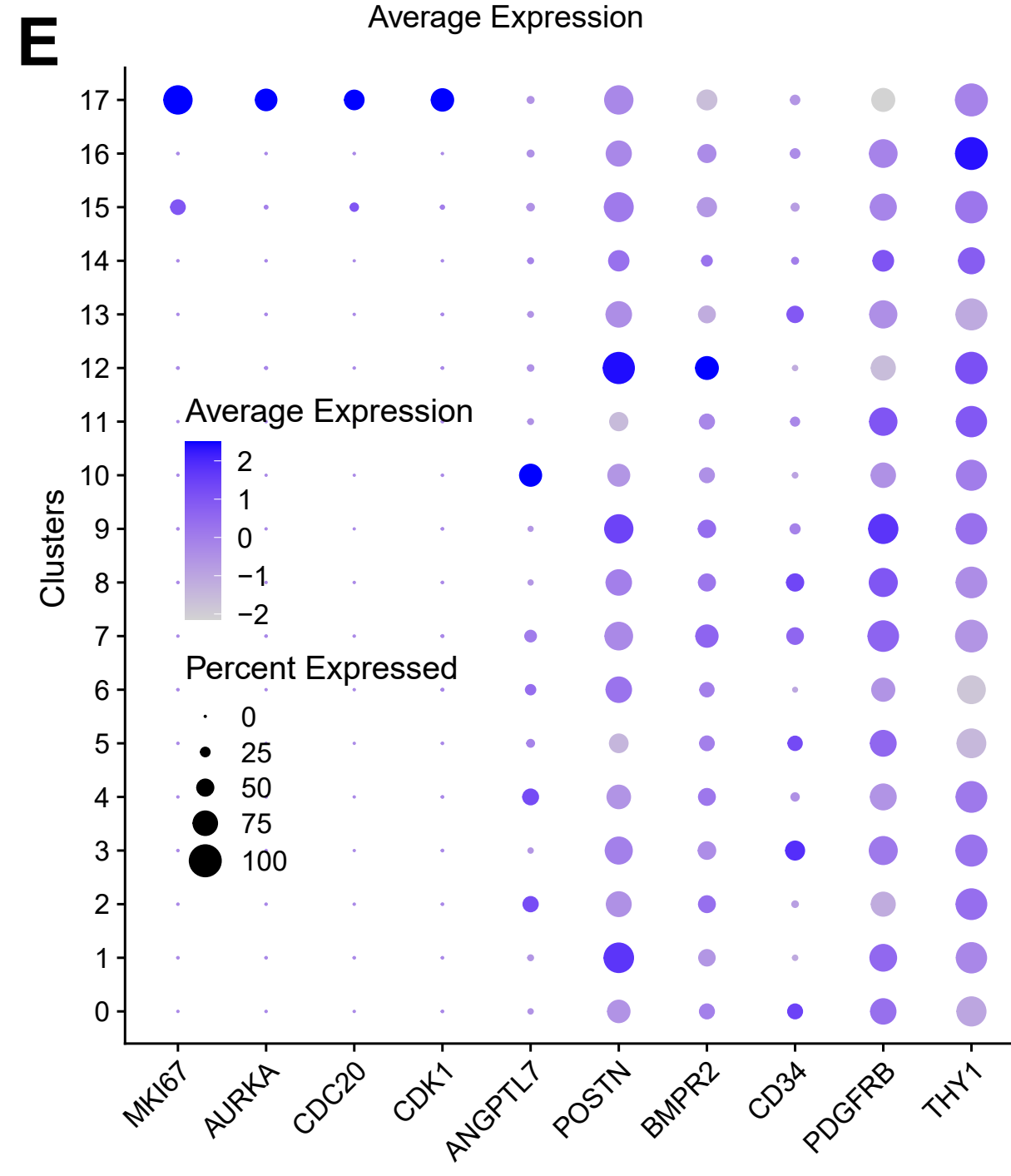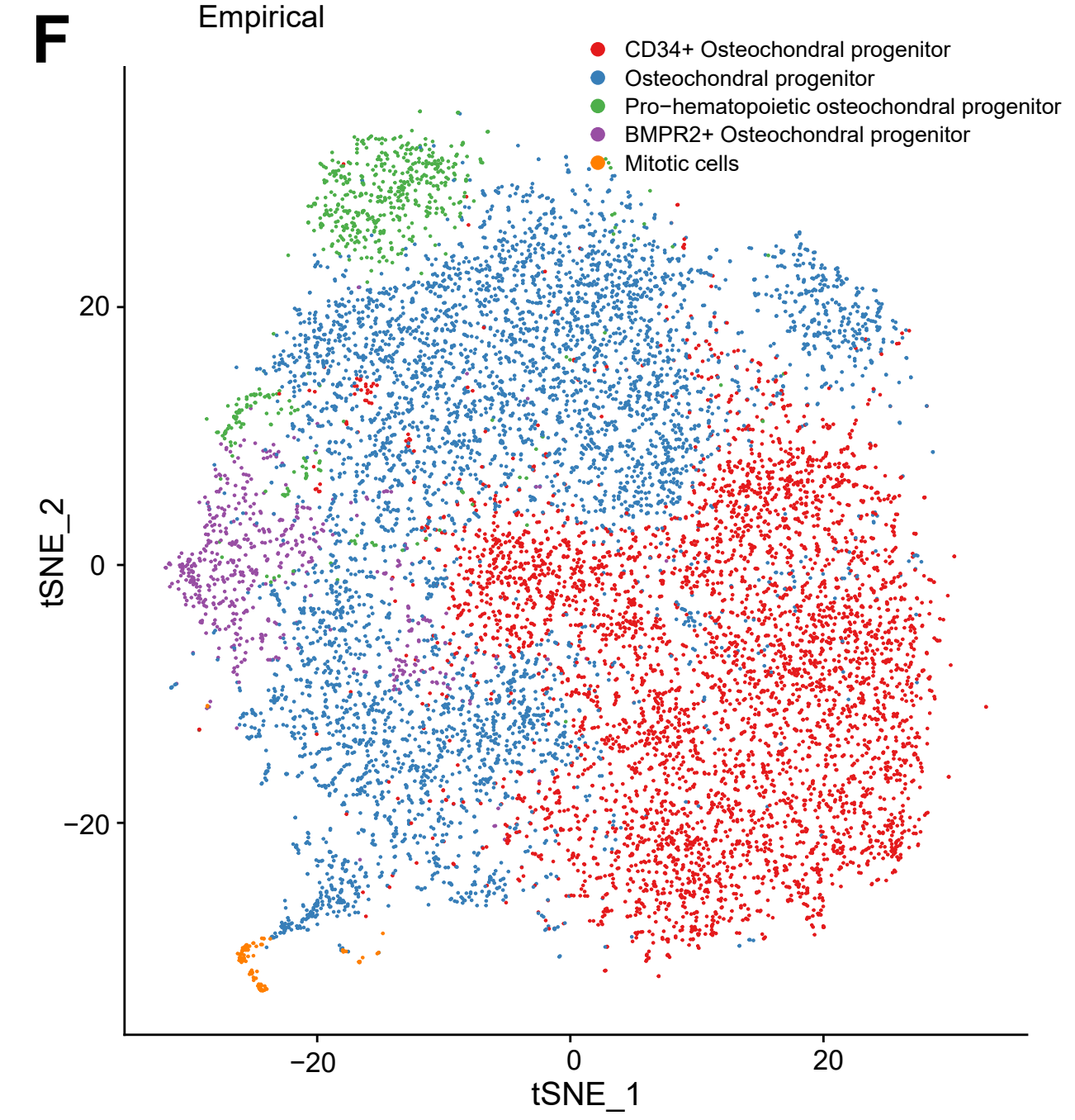

Supplement: Supplemental Information 1 — (A) Cell quality indicators. (B) Mean expression values and standard deviations of genes. (C) Principal component significance. (D) Cellular tSNE descending clustering. (E) Mean expression values and expression proportions of marker genes in clustered subgroups. (F) Distribution of different cell types includes: CD34+ osteochondral progenitor (n = 4563), osteochondral progenitor (n = 5316), pro-hematopoietic osteochondral progenitor (n = 600), BMPR2+ osteochondral progenitor (n = 534) and mitotic cells. (Related to Figs. 1A & 1B) [file peerj-10-13722-s001.pdf]

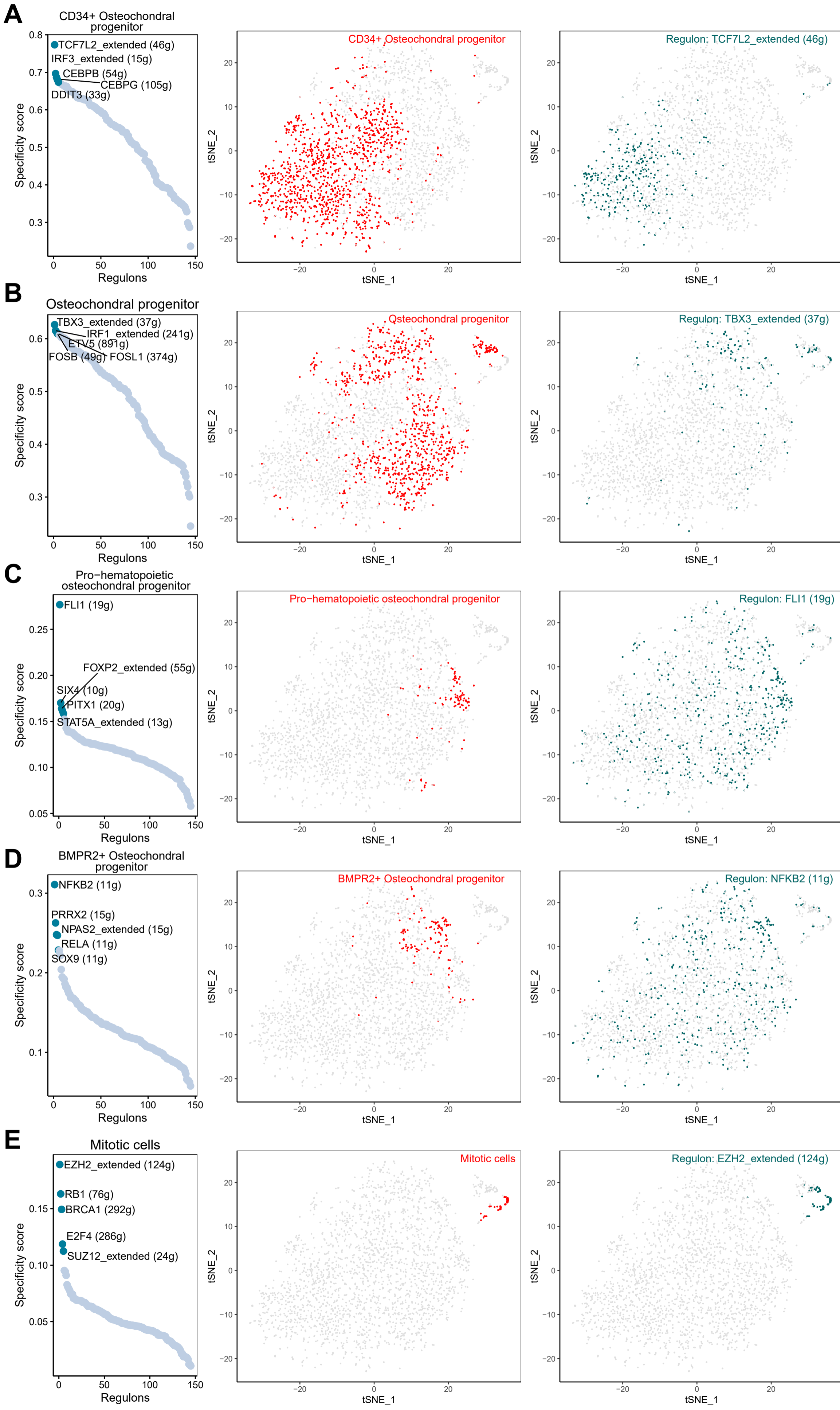

Supplement: Supplemental Information 2 — (A) The left panel indicates regulators activated in VEGF+ cells sorted according to specificity scoring with CD34+ osteochondral progenitor, with the top 5 regulators considered relevant. The middle panel highlights CD34+ osteochondral progenitor in all cells. The right panel is the fraction with the most specific regulator of CD34+ osteochondral progenitor, TCF7L2, activated in all VEGF+ cells. Like (B–E). (Related to Figs. 1A & 1B) [file peerj-10-13722-s002.pdf]

**A**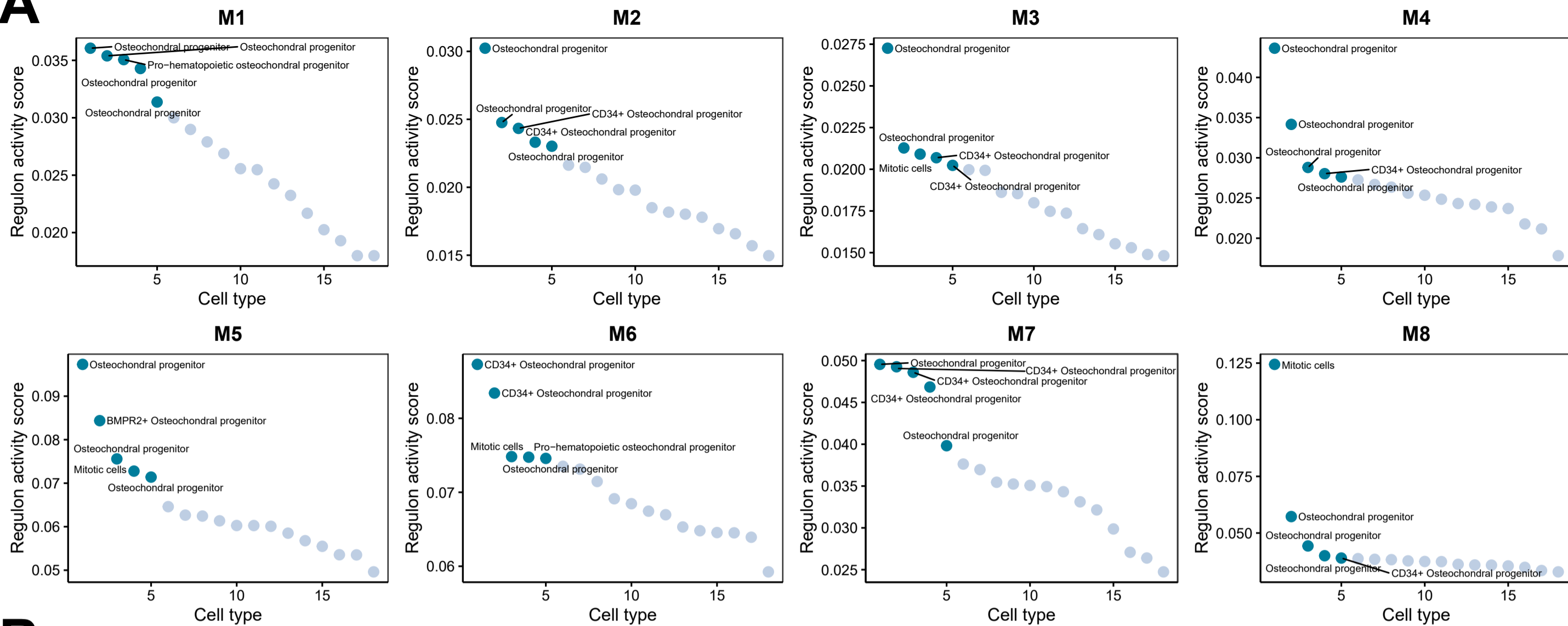**B**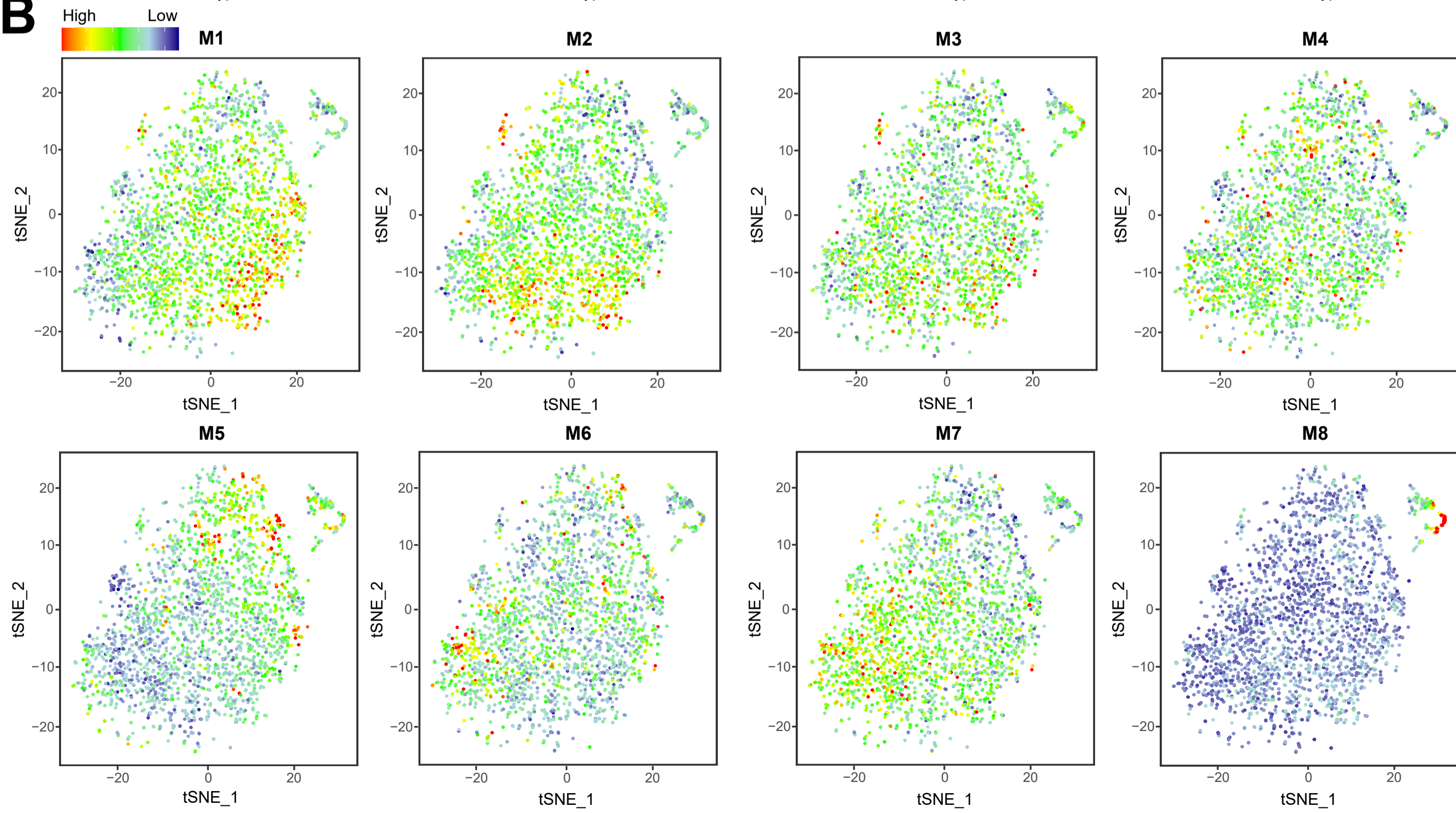

Supplement: Supplemental Information 3 — (A) Cell types were ranked according to their activity scoring with regulatory modules. (B) Activity scoring of regulatory modules in VEGF+ cells. (Related to Fig. 2) [file peerj-10-13722-s003.pdf]

A

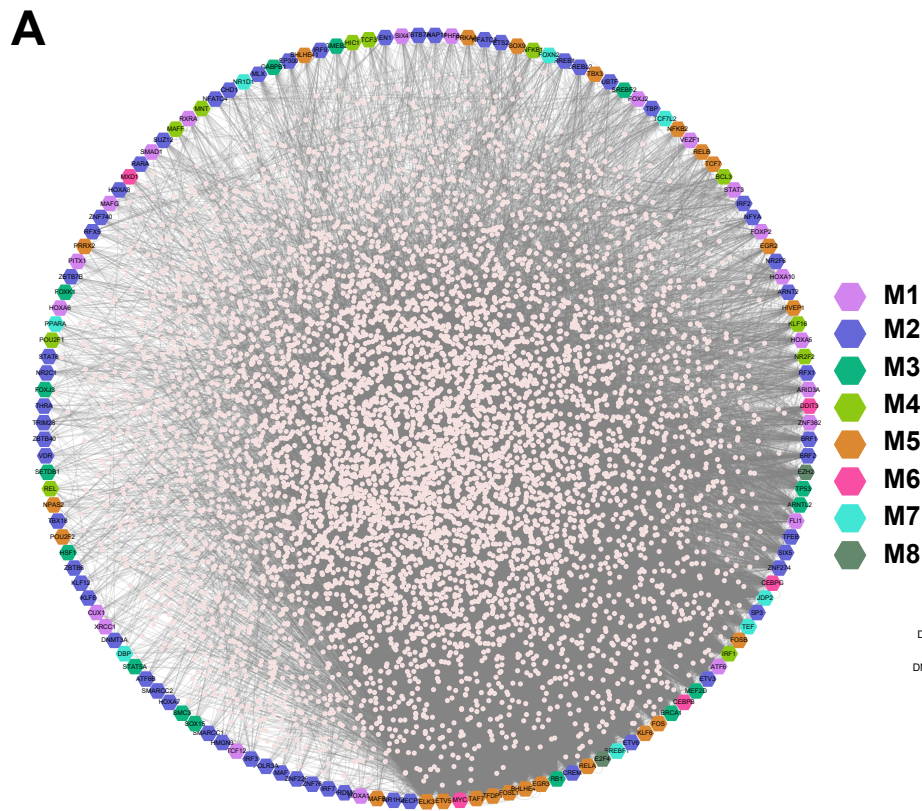

B

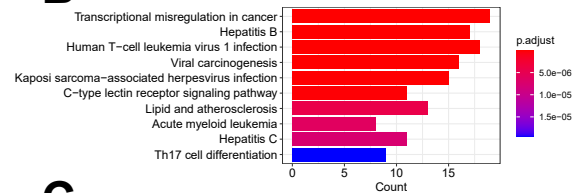

C

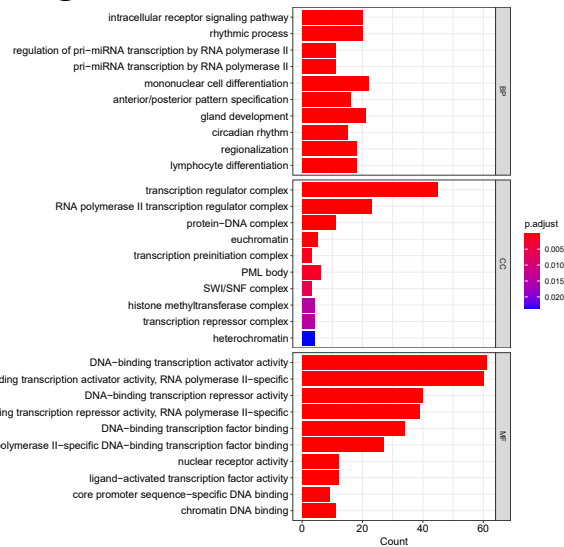

Supplement: Supplemental Information 4 — (A) Transcription factor regulatory networks activated in VEGF+ cells are shown. Deformed dots indicate regulatory factors, and pink dots indicate target genes. (B)Transcription factors activated in cells significantly enriched in the KEGG pathway of the top 10 pathways. (C) GSEA analysis showing the different pathways significantly upregulated in BP, MF, and CC. (Related to Fig. 2) [file peerj-10-13722-s004.pdf]

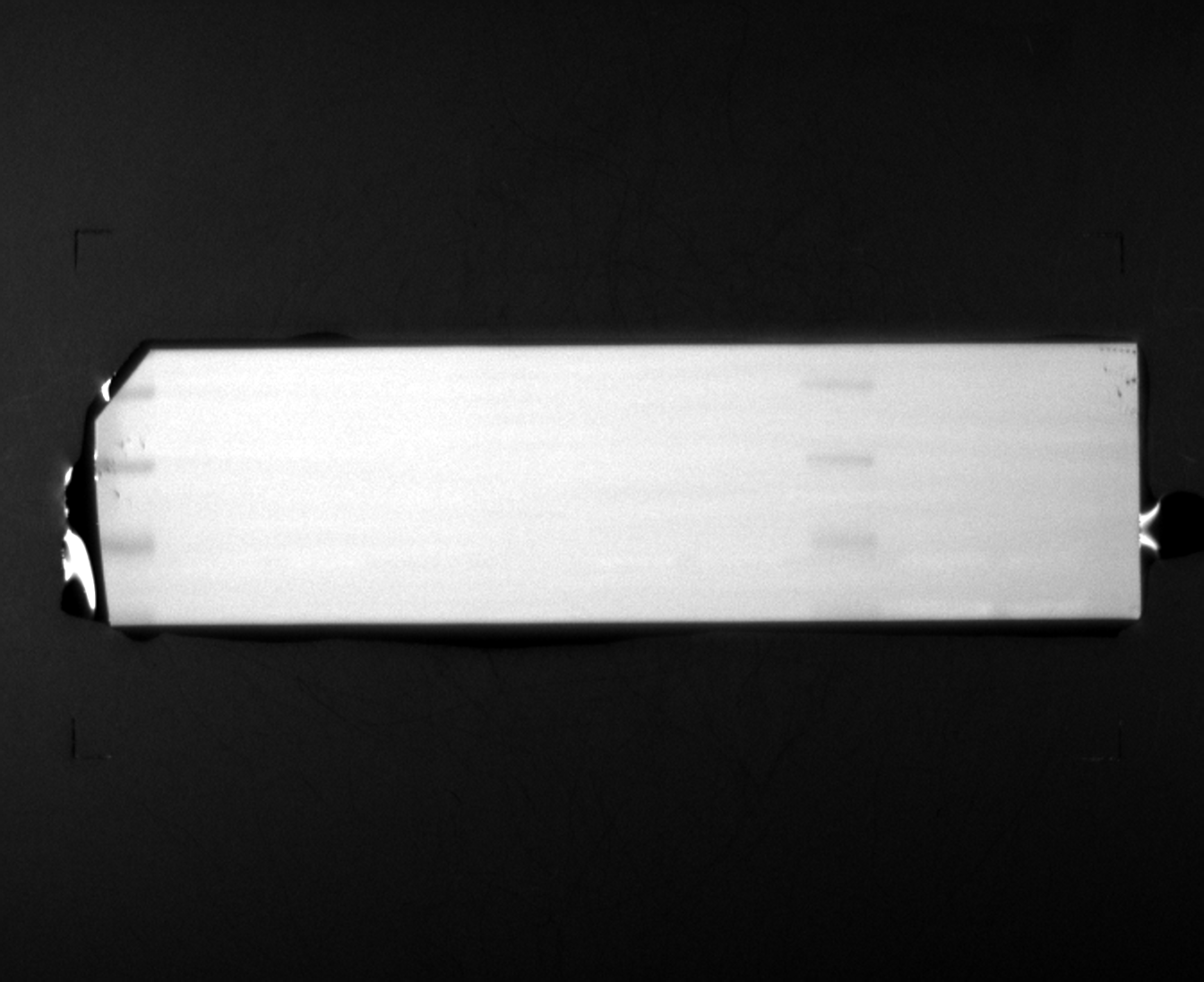

Supplement: Supplemental Information 6 [file peerj-10-13722-s006.zip › WB/GAPDH/0.Tif]

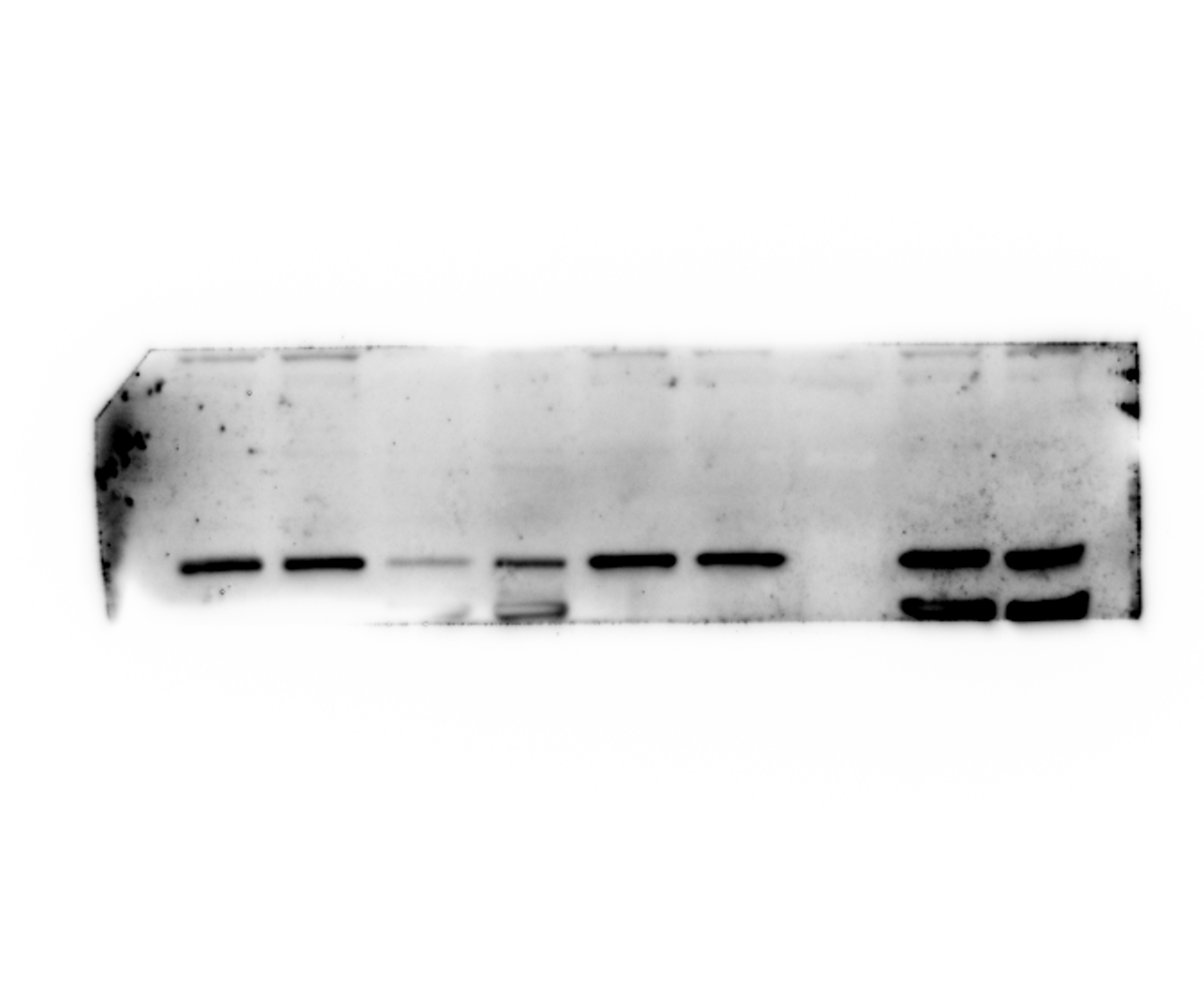

Supplement: Supplemental Information 6 [file peerj-10-13722-s006.zip › WB/GAPDH/10.Tif]

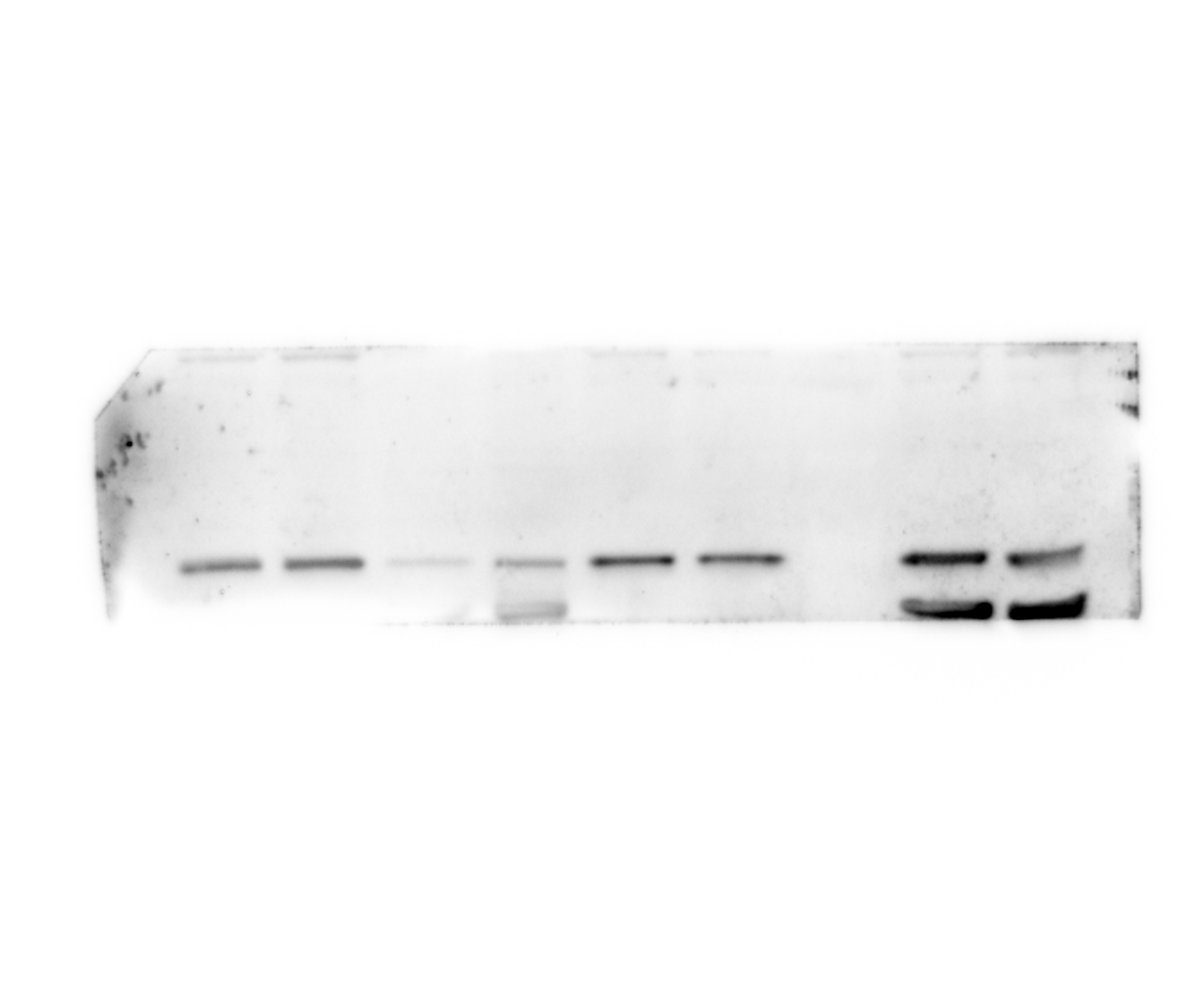

Supplement: Supplemental Information 6 [file peerj-10-13722-s006.zip › WB/GAPDH/2.Tif]

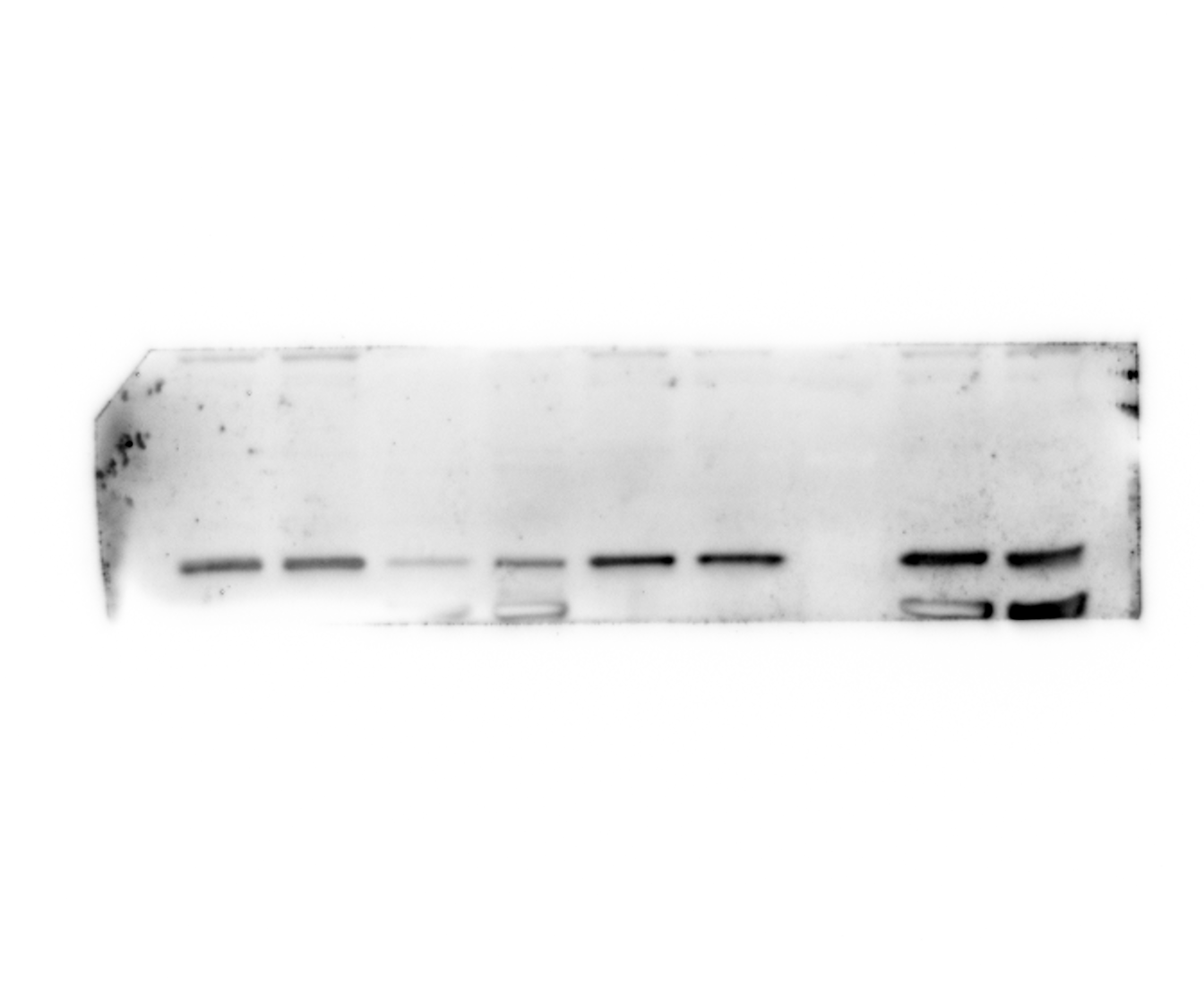

Supplement: Supplemental Information 6 [file peerj-10-13722-s006.zip › WB/GAPDH/3.Tif]

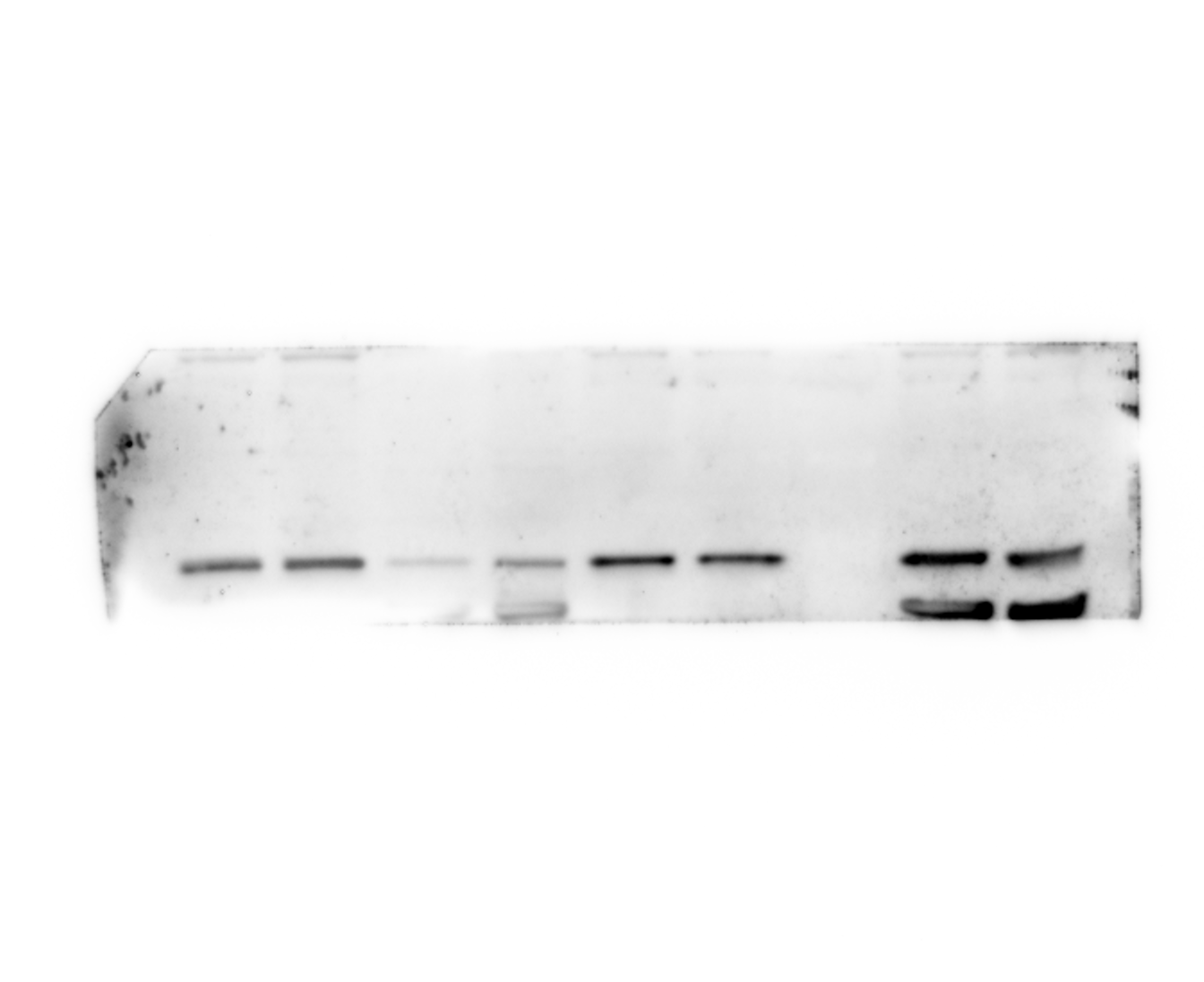

Supplement: Supplemental Information 6 [file peerj-10-13722-s006.zip › WB/GAPDH/5.Tif]

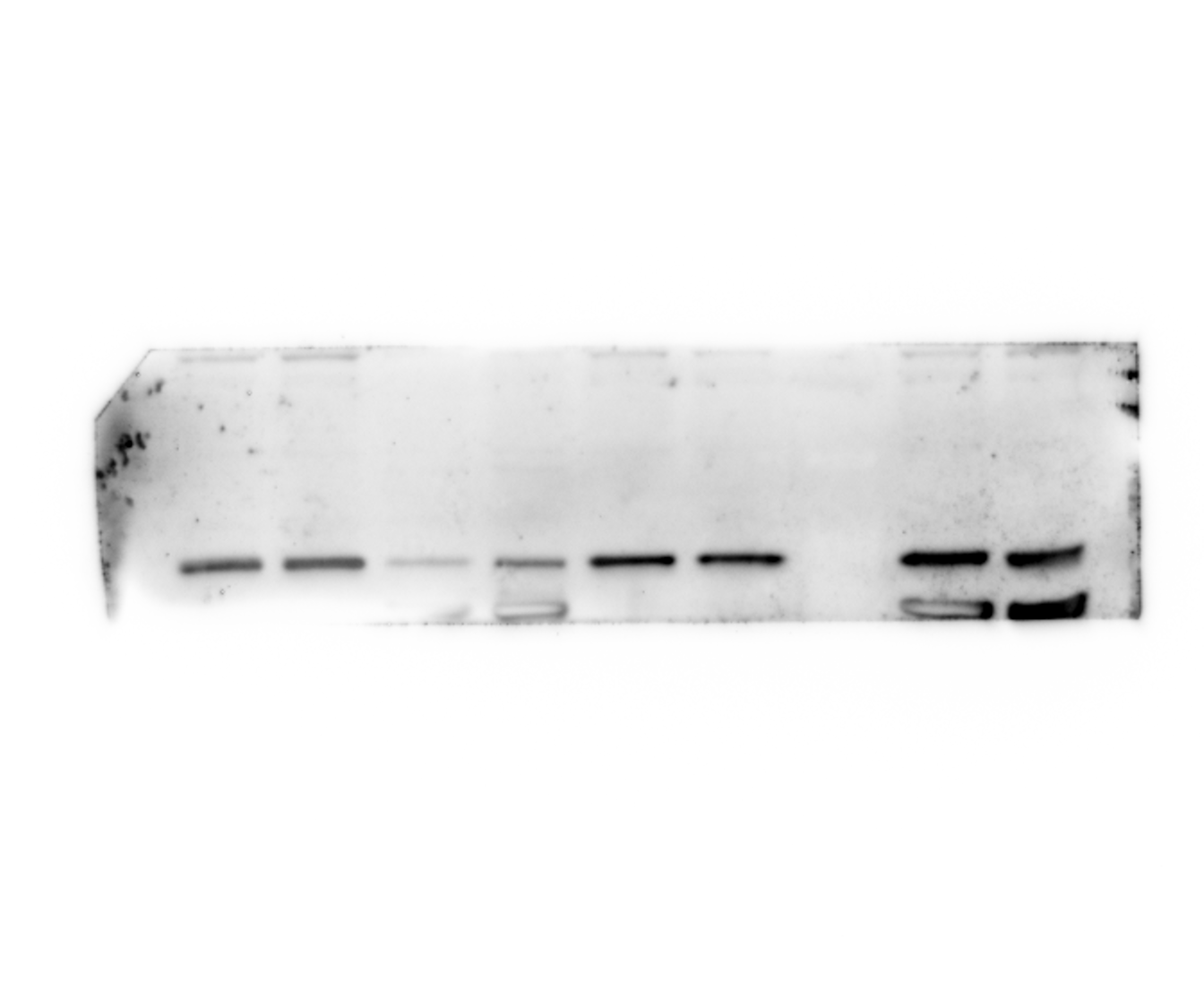

Supplement: Supplemental Information 6 [file peerj-10-13722-s006.zip › WB/GAPDH/7.Tif]

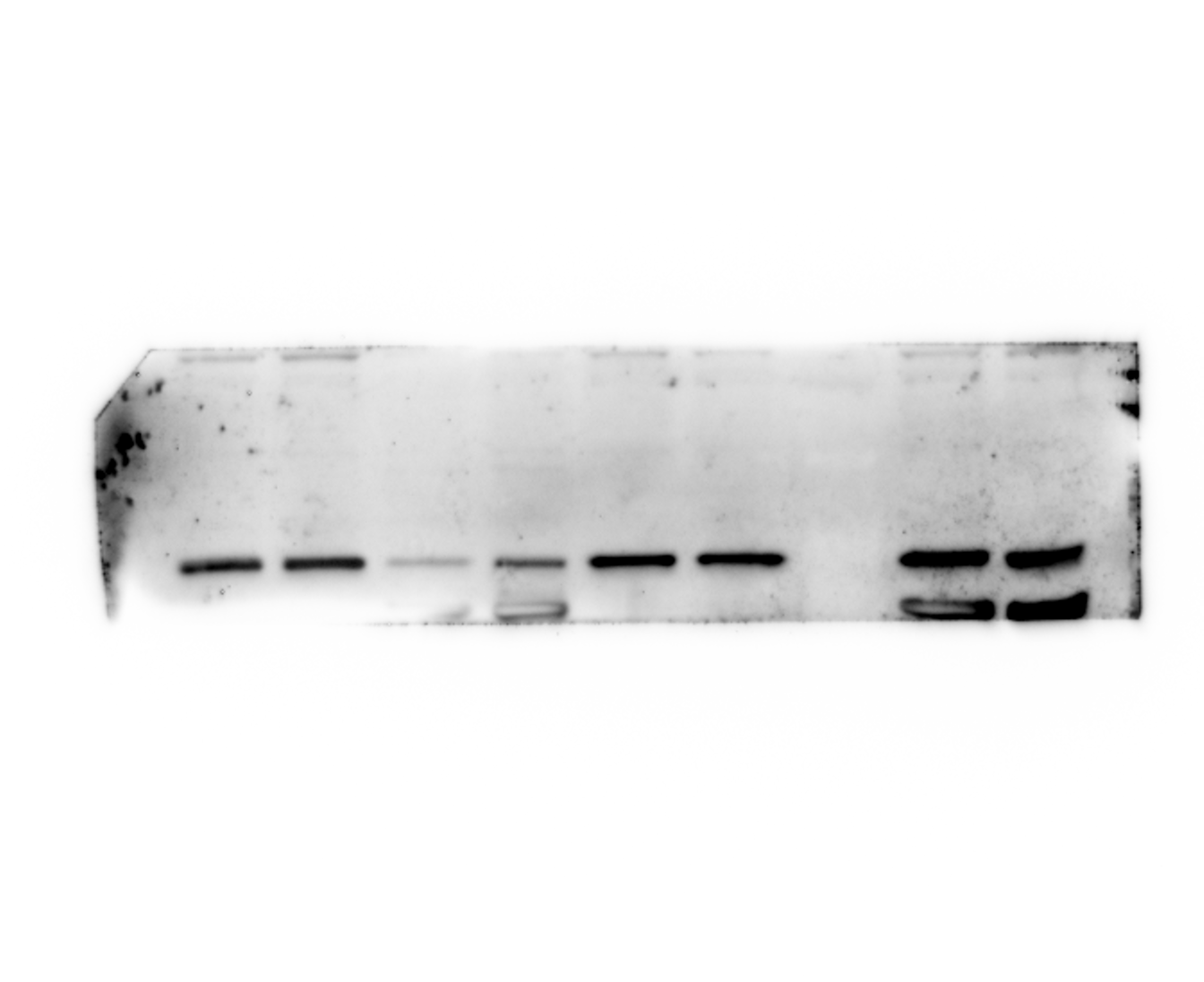

Supplement: Supplemental Information 6 [file peerj-10-13722-s006.zip › WB/GAPDH/8.Tif]

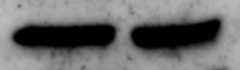

Supplement: Supplemental Information 6 [file peerj-10-13722-s006.zip › WB/GAPDH/GAPDH.tif]

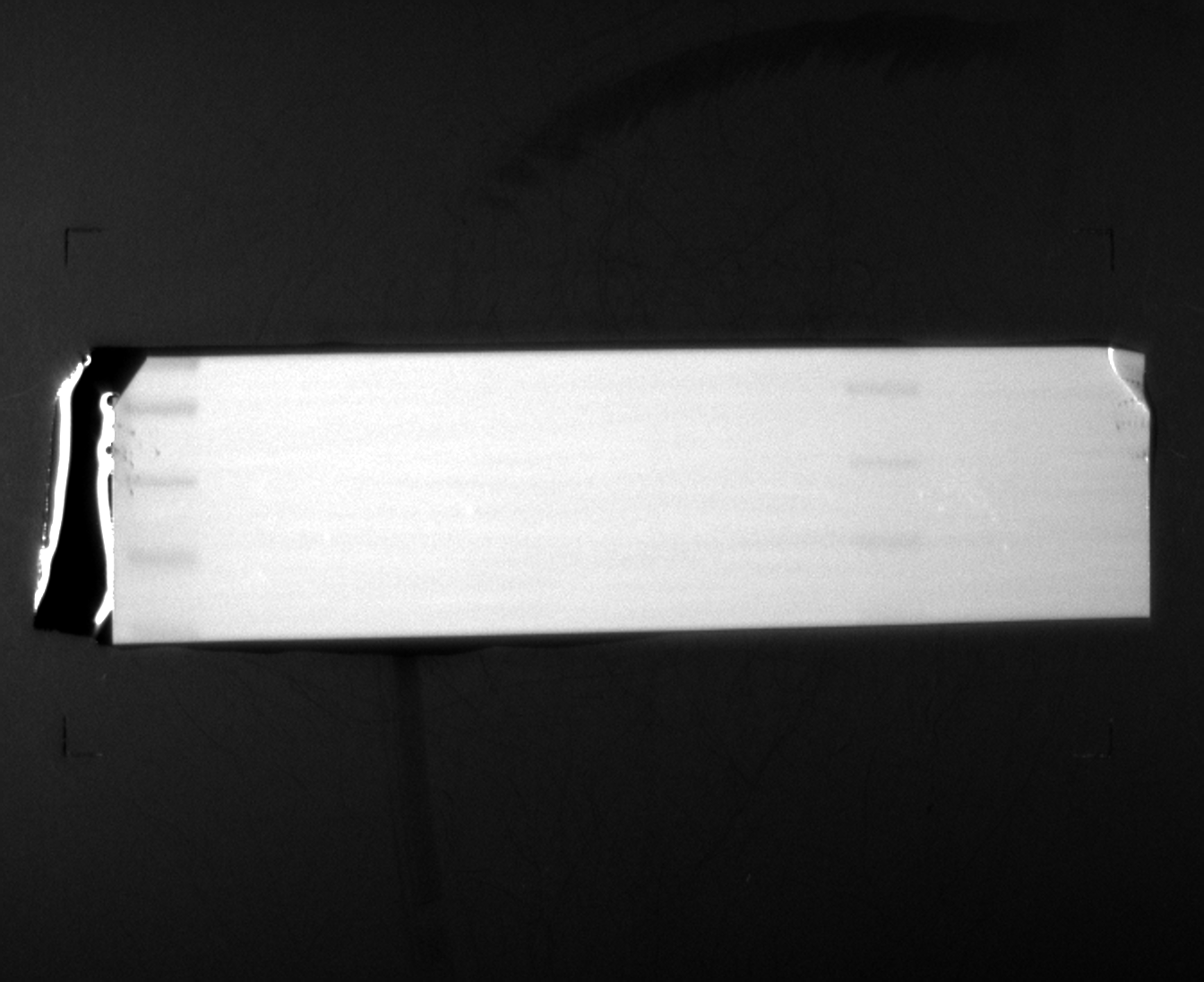

Supplement: Supplemental Information 6 [file peerj-10-13722-s006.zip › WB/VEGFA/0.Tif]

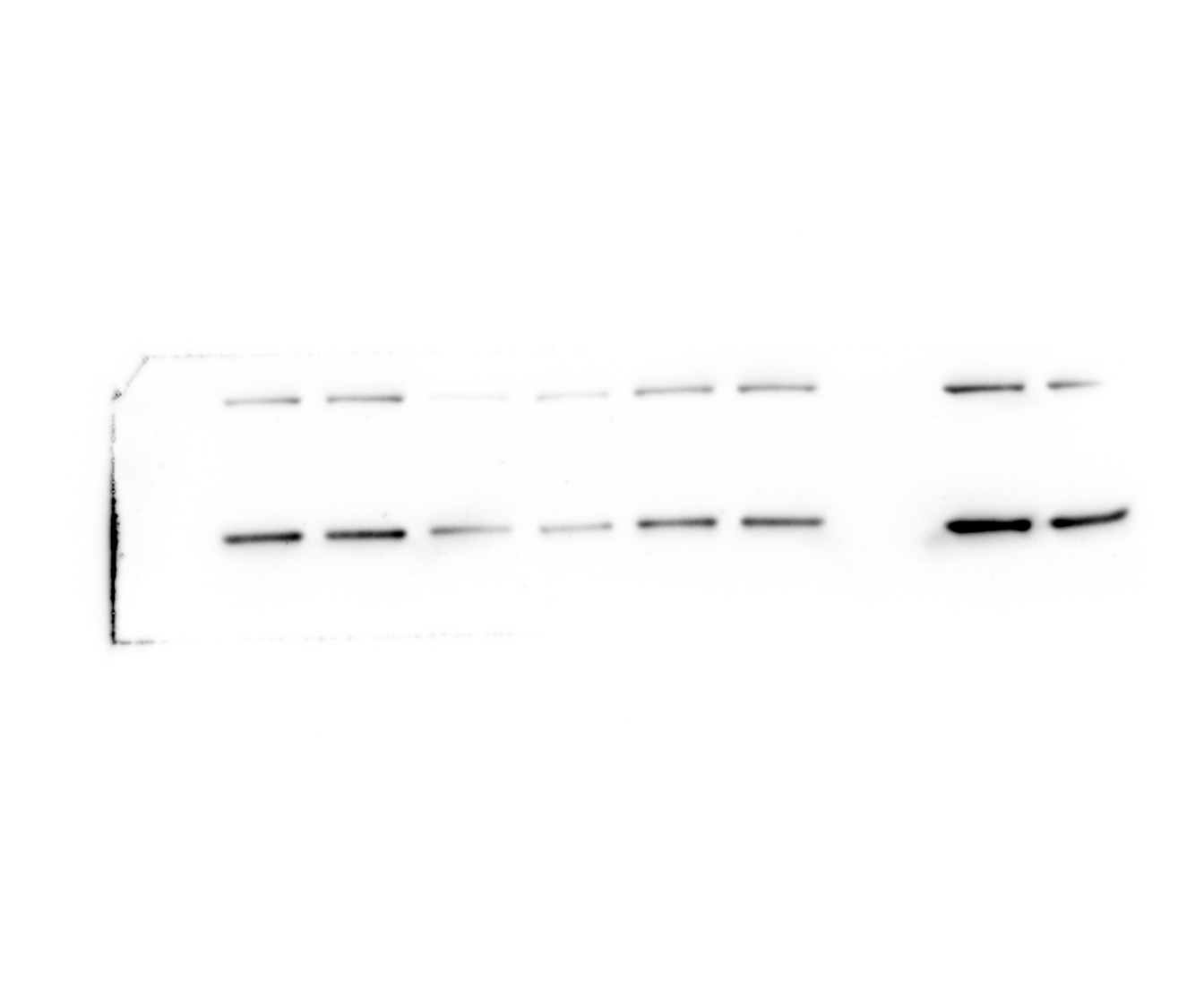

Supplement: Supplemental Information 6 [file peerj-10-13722-s006.zip › WB/VEGFA/10.Tif]

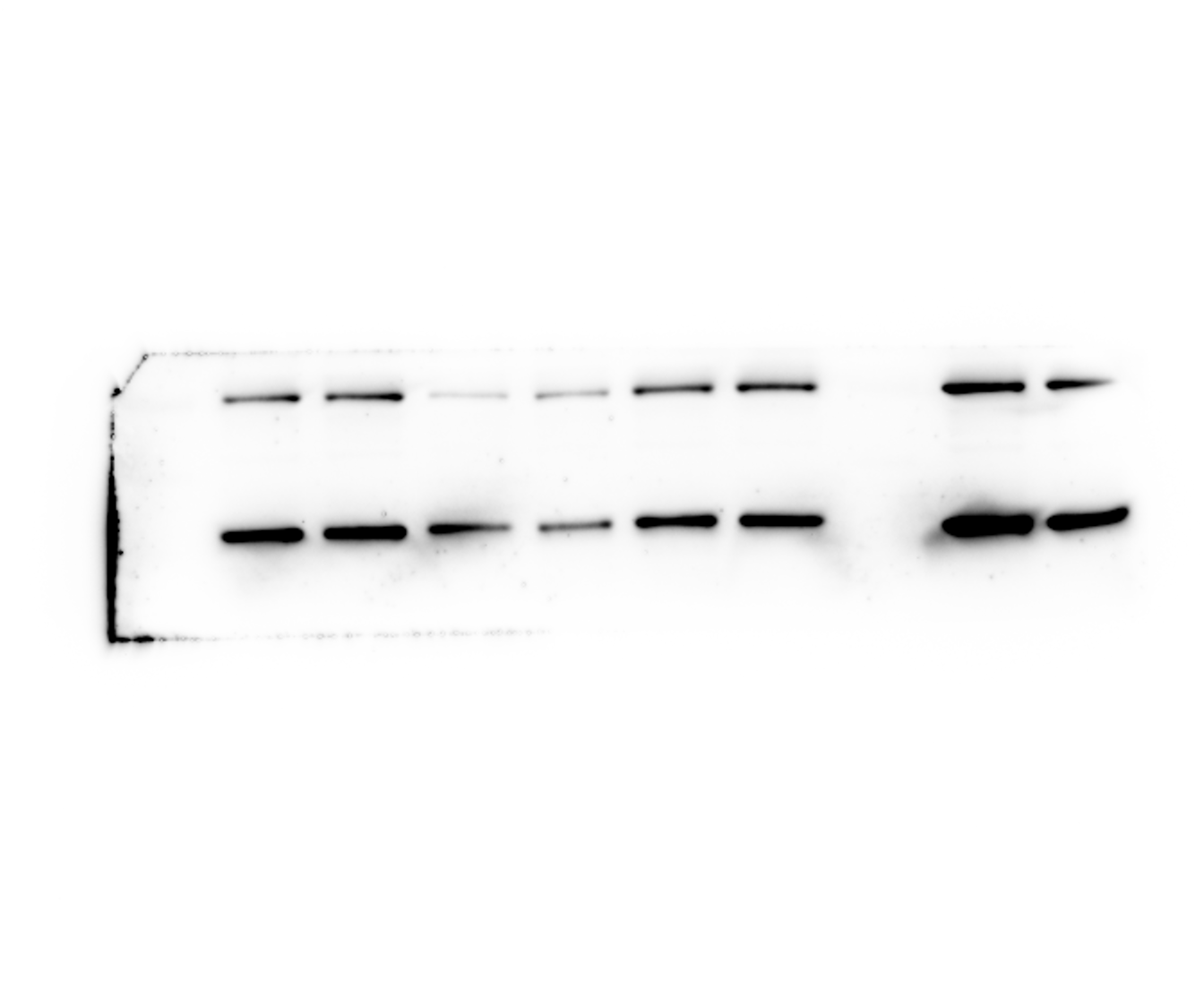

Supplement: Supplemental Information 6 [file peerj-10-13722-s006.zip › WB/VEGFA/100.Tif]

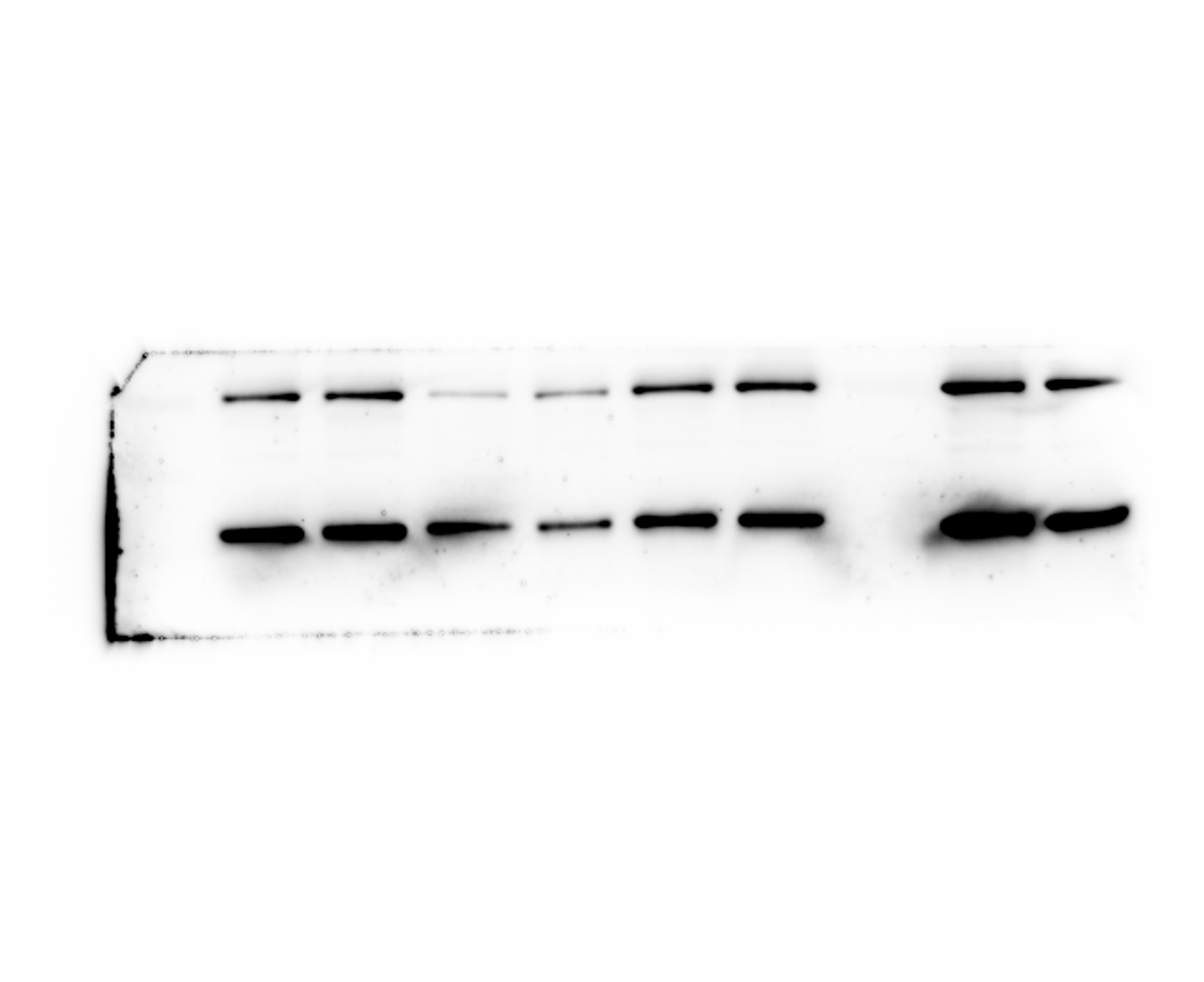

Supplement: Supplemental Information 6 [file peerj-10-13722-s006.zip › WB/VEGFA/150.Tif]

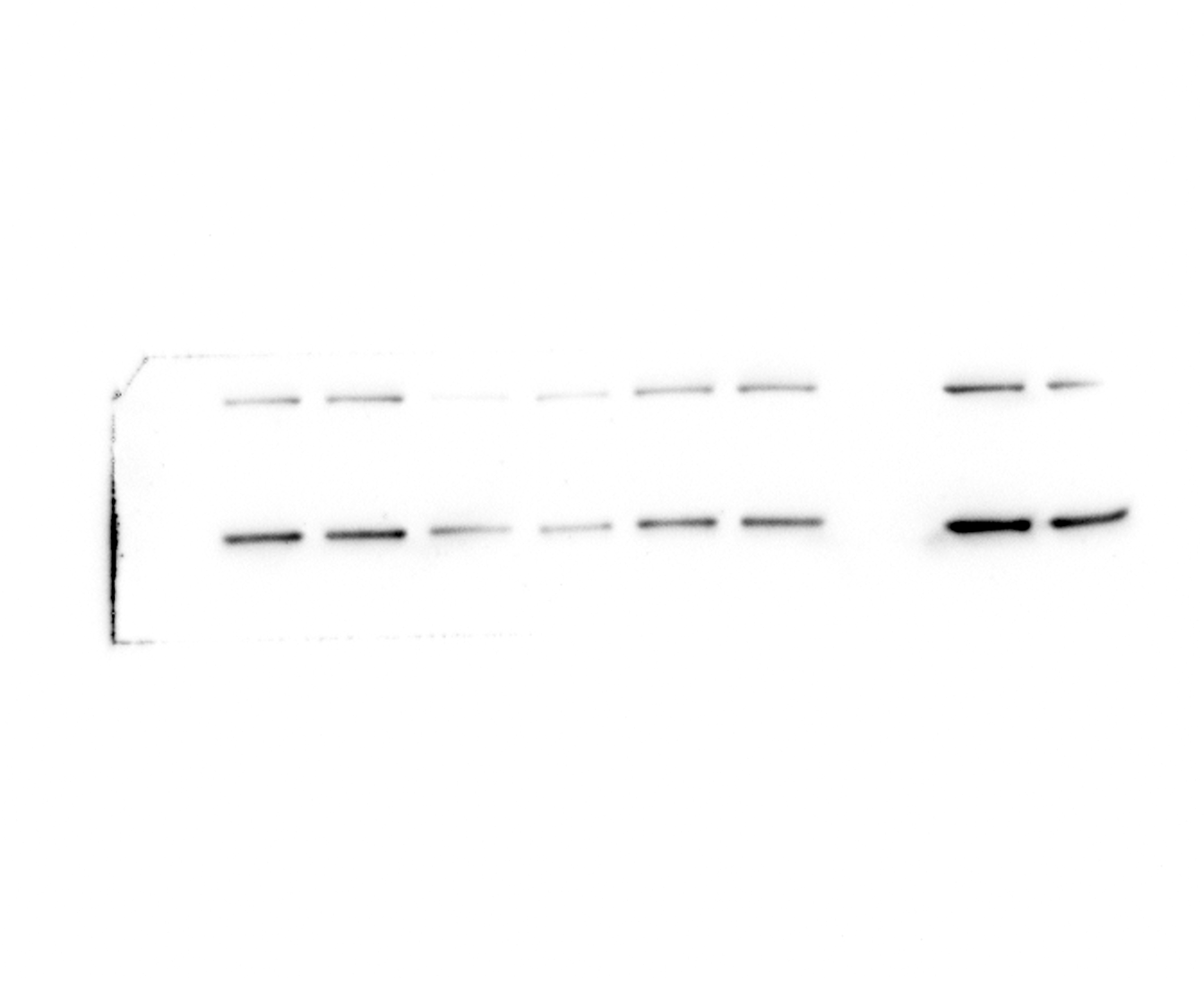

Supplement: Supplemental Information 6 [file peerj-10-13722-s006.zip › WB/VEGFA/3.Tif]

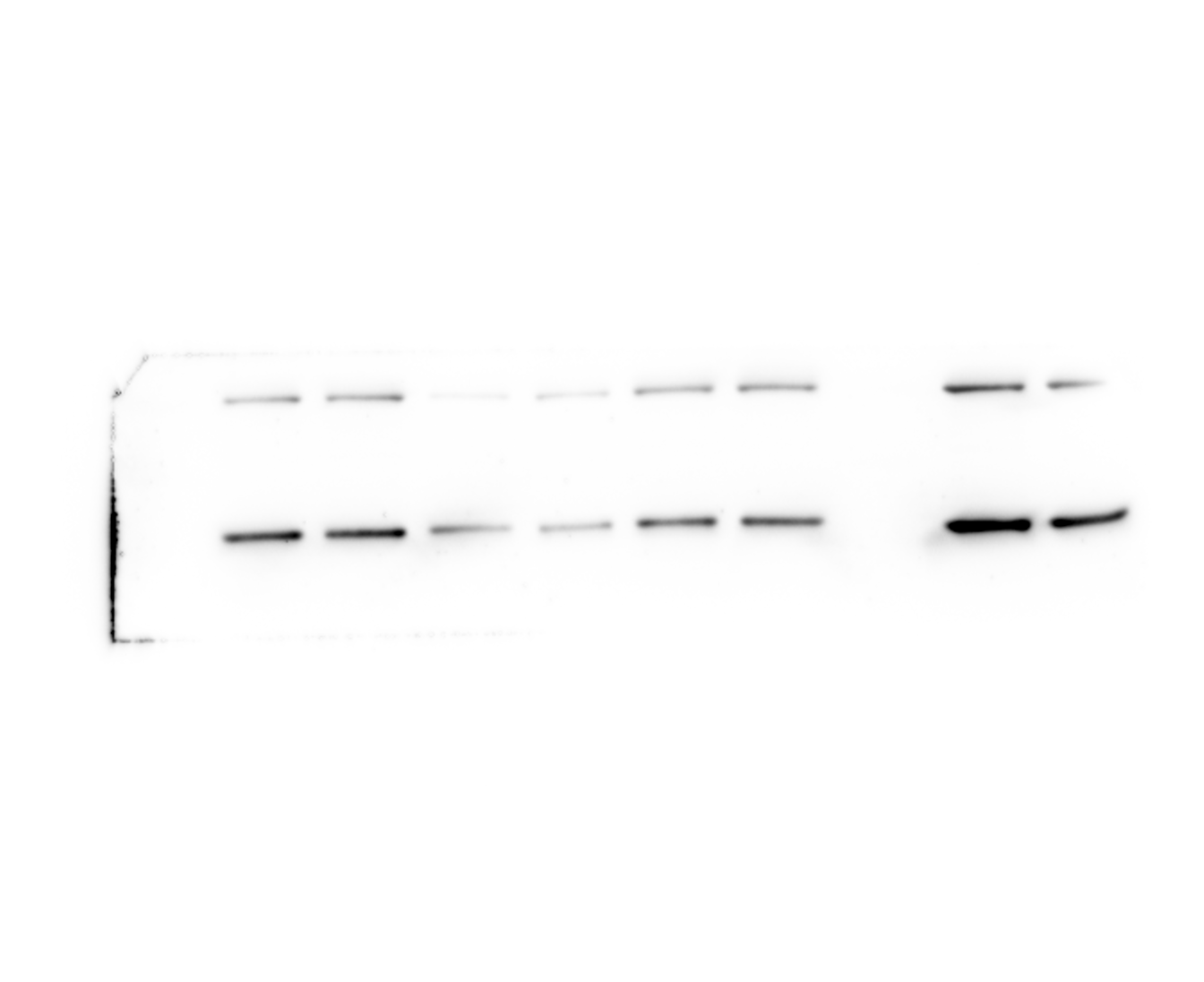

Supplement: Supplemental Information 6 [file peerj-10-13722-s006.zip › WB/VEGFA/30.Tif]

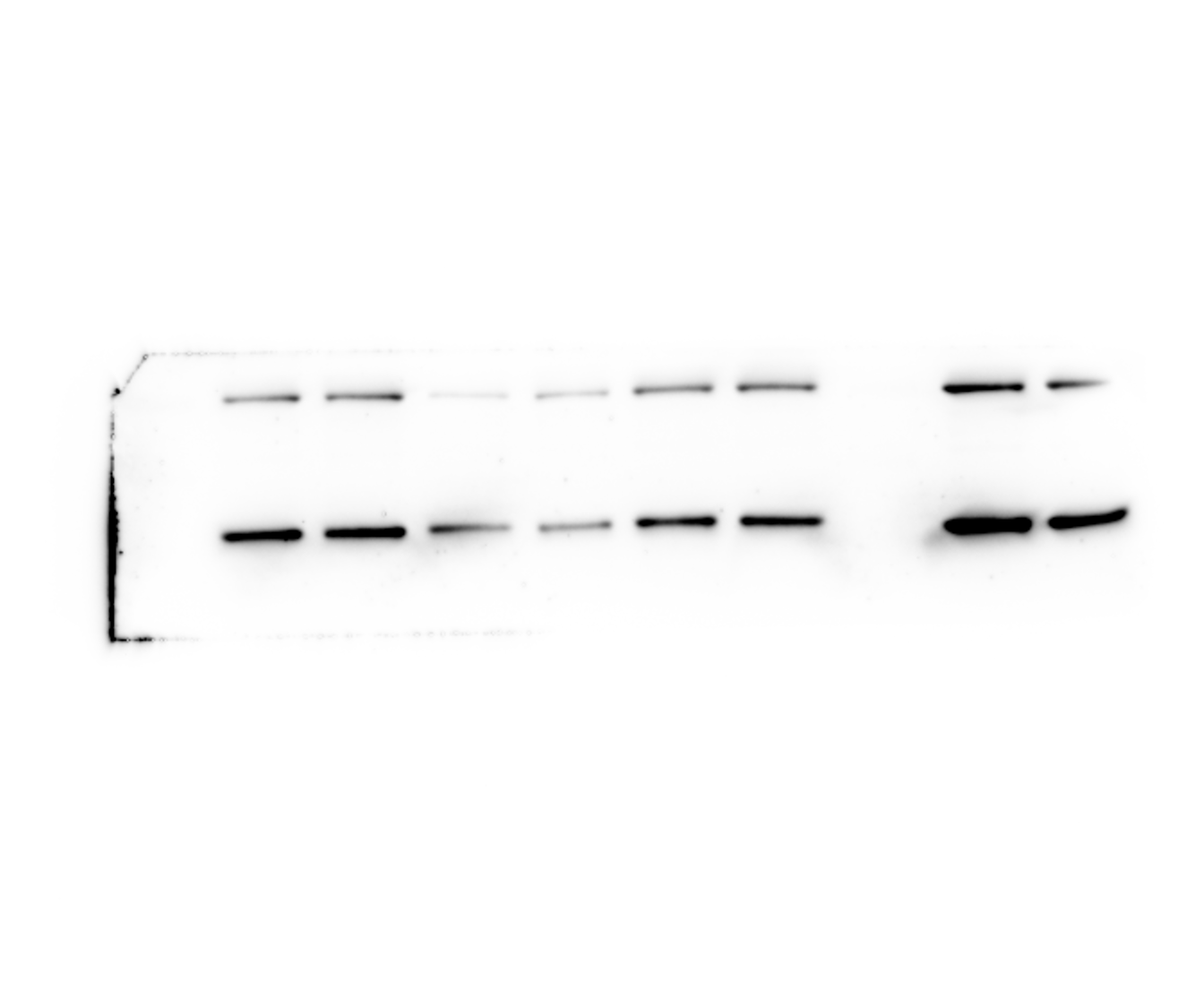

Supplement: Supplemental Information 6 [file peerj-10-13722-s006.zip › WB/VEGFA/60.Tif]

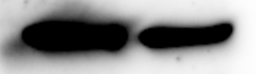

Supplement: Supplemental Information 6 [file peerj-10-13722-s006.zip › WB/VEGFA/VEGFA.tif]
